# Middle Eocene greenhouse warming facilitated by diminished weathering feedback

**DOI:** 10.1038/s41467-018-05104-9

**Published:** 2018-07-23

**Authors:** Robin van der Ploeg, David Selby, Marlow Julius Cramwinckel, Yang Li, Steven M. Bohaty, Jack J. Middelburg, Appy Sluijs

**Affiliations:** 1 Department of Earth Sciences, Faculty of Geosciences, Utrecht University, Utrecht, 3584 CB The Netherlands; 2 Department of Earth Sciences, Durham University, Durham, DH1 3LE UK; 3 State Key Laboratory of Geological Processes and Mineral Resources, School of Earth Resources, China University of Geosciences, Wuhan, 430074 Hubei China; 4 Department of Geology and Geophysics, Yale University, New Haven, Connecticut 06511 USA; 5 State Key Laboratory of Lithospheric Evolution, Institute of Geology and Geophysics, Chinese Academy of Sciences, Beijing, 10029 China; 6 Ocean and Earth Science, National Oceanography Centre, University of Southampton Waterfront Campus, Southampton, SO14 3ZH UK

**Keywords:** Carbon cycle, Element cycles, Climate and Earth system modelling, Palaeoceanography, Palaeoclimate

## Abstract

The Middle Eocene Climatic Optimum (MECO) represents a ~500-kyr period of global warming ~40 million years ago and is associated with a rise in atmospheric CO_2_ concentrations, but the cause of this CO_2_ rise remains enigmatic. Here we show, based on osmium isotope ratios (^187^Os/^188^Os) of marine sediments and published records of the carbonate compensation depth (CCD), that the continental silicate weathering response to the inferred CO_2_ rise and warming was strongly diminished during the MECO—in contrast to expectations from the silicate weathering thermostat hypothesis. We surmise that global early and middle Eocene warmth gradually diminished the weatherability of continental rocks and hence the strength of the silicate weathering feedback, allowing for the prolonged accumulation of volcanic CO_2_ in the oceans and atmosphere during the MECO. These results are supported by carbon cycle modeling simulations, which highlight the fundamental importance of a variable weathering feedback strength in climate and carbon cycle interactions in Earth’s history.

## Abstract (web summary)

Silicate rock weathering represents a negative feedback mechanism that regulates atmospheric CO_2_ levels on geological timescales. Here, the authors show that a diminished silicate weathering feedback may have set the stage for greenhouse warming and ocean acidification during the Middle Eocene, ~40 million years ago.

## Introduction

The chemical weathering of silicate rocks represents a negative feedback mechanism that is generally considered to modulate atmospheric CO_2_ levels and Earth’s climate on geological timescales^1,2^. This phenomenon has been studied for various carbon cycle perturbations and episodes of global warming in the geological past, including Pleistocene deglaciations, the Paleocene-Eocene Thermal Maximum (PETM; ~56 Ma), and the Cretaceous and Jurassic Oceanic Anoxic Events (OAEs), mainly through the application of isotope ratios of marine sediments that are sensitive to shifts in weathering fluxes or compositions on the appropriate timescales^3–5^. For many of these phases, it is now relatively well established that enhanced continental weathering contributed to CO_2_ drawdown and climatic recovery^4,6,7^. However, the available data spanning the Middle Eocene Climatic Optimum (MECO; ~40 Ma) pose questions regarding the functioning of the weathering feedback^8^. Over a period of ~500 kyr, global ocean temperatures rose gradually by up to ~5 °C in association with an increase in atmospheric CO_2_ concentrations, sourced from a reservoir with a stable carbon isotopic composition (δ^13^C) close to that of the ocean^9–13^. Importantly, the inferred rise in atmospheric CO_2_ and temperature over ~500 kyr during the MECO should have led to increased weathering and alkalinity supply to the oceans, but reconstructions show that the oceans acidified^8,10^. Therefore, reconstructing the global weathering response during the MECO is instrumental to improving our fundamental understanding of carbon cycle dynamics on such intermediate timescales of ~500 kyr^8^.

A promising proxy to reconstruct changes in continental weathering during the MECO is the osmium isotope ratio of marine sediments at the time of deposition (^187^Os/^188^Os_initial_, or Os_*i*_)^14,15^. The ^187^Os/^188^Os ratio of the global ocean is governed by the relative input of radiogenic Os (^187^Os/^188^Os = ~1.4) through continental weathering of ancient crustal rocks, and the relative input of unradiogenic Os (^187^Os/^188^Os = 0.13) through hydrothermal activity at mid-ocean ridges and weathering of fresh mantle-derived rocks, with additional contributions from extraterrestrial sources^14^. Osmium is a quasi-conservative element that is well-mixed in the ocean, and has a short oceanic residence time (generally ~10^4^ yr in the open ocean, but residence times of ~10^3^ yr have been inferred for very restricted settings)^14,16^. Variations in the ^187^Os/^188^Os ratio of seawater are thus indicative of changes in continental weathering relative to the other sources on timescales shorter than, or similar to, climate and carbon cycle processes such as greenhouse warming, ocean acidification, and carbonate compensation^14,15^. Seawater Os is incorporated in the metalliferous and organic phases of marine sediments without isotopic fractionation, and remains a closed isotopic system from the time of deposition^17–19^. As such, Os_*i*_ values are calculated on the basis that radiogenic ^187^Os ingrowth is derived solely from post-depositional ^187^Re (rhenium) decay. Shifts to higher (radiogenic) Os_*i*_ values, which are attributed to a global increase in continental silicate weathering rates, have been recorded for carbon cycle perturbations such as the Toarcian OAE and the PETM and Eocene Thermal Maximum 2 (ETM2) transient global warming events^5,15,20^.

A second parameter that is often used to constrain changes in continental weathering is the carbonate compensation depth (CCD). The CCD is the depth in the oceans at which carbonate delivery is balanced by carbonate dissolution, and is modulated by the interplay of volcanic CO_2_ degassing, the weathering of silicate rocks and organic-rich sediments on land, and the burial of marine carbonates and organic carbon^21^. As such, changes in the position of the CCD as reflected in sediments play a crucial role in reconstructions of carbon cycle change, both on multi-million year timescales and during transient perturbations such as the MECO^22^.

In this study, we present Os_*i*_ records of marine sediments from three locations in different ocean basins in combination with a compilation of published CCD records^8^ to reconstruct global changes in continental weathering during the MECO. Rather than an Os_*i*_ increase expected from globally enhanced weathering, we document a modest global Os_*i*_ decrease during the MECO that may represent an episode of enhanced volcanism and/or associated basalt weathering. In fact, prolonged CCD shoaling precludes an increase in total continental weathering rates in response to CO_2_ rise and greenhouse warming. We employ a series of simulations with the carbon cycle model LOSCAR^23^ together with an independent osmium cycle model to demonstrate that this combination of observations can only be successfully reconciled on MECO timescales by invoking enhanced volcanism together with a diminished continental weathering feedback. Finally, we surmise that such a reduced silicate weathering feedback may have resulted from a progressive decrease in the weatherability of the continents during the Eocene. A variable silicate weathering feedback strength may have been important for other enigmatic climate and carbon cycle perturbations in Earth’s history.

## Results

### Middle Eocene osmium isotope records

**Fig. 1:**
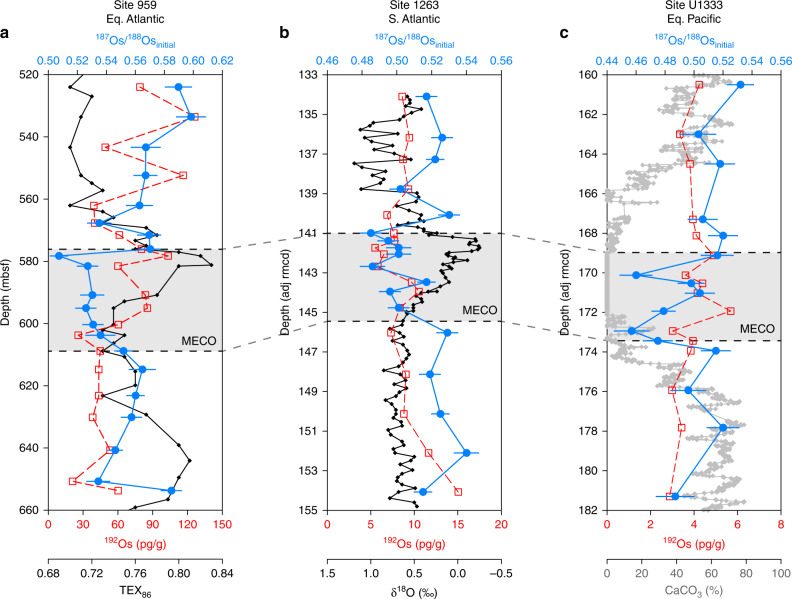
Os_*i*_ values (in blue) and ^192^Os concentrations (in red) for the analyzed middle Eocene sediments from the three different sites. **a** ODP Site 959; **b** ODP Site 1263; **c** IODP Site U1333. The MECO interval is defined based on TEX_86_ values for Site 959 (in black; Cramwinckel et al.^13^) and bulk carbonate stable oxygen isotope ratios (δ^18^O) for Site 1263 (in black; Bohaty et al.^10^). The MECO is characterized by low carbonate content at Site U1333 (in grey; Westerhold et al.^84^). The error bars indicate fully propagated analytical uncertainties (2*σ*)

**Fig. 2:**
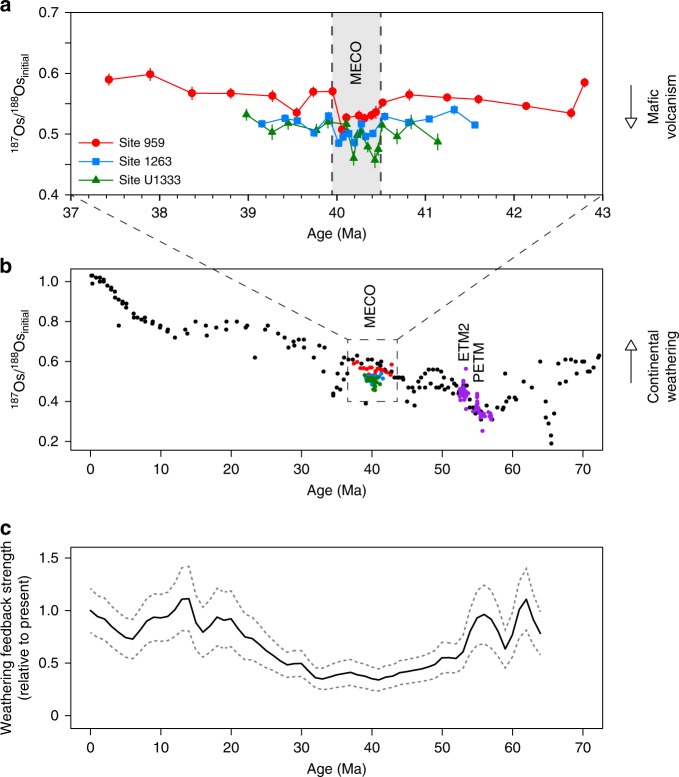
Comparison of Os_*i*_ records from the MECO with Os_*i*_ records from the PETM and ETM2, shown against the overall Os_*i*_ evolution of the Cenozoic and the relative weathering feedback strength of the Cenozoic. **a** MECO data from Site 959 (in red), Site 1263 (in blue) and Site U1333 (in green) plotted against age (GTS2012)^74^. See Methods for discussion of the age models for the study sites. **b** MECO data from Sites 959, 1263, and U1333 (this study); PETM and ETM2 data from DSDP Site 549 (in purple) as published in Peucker-Ehrenbrink & Ravizza^15^; Cenozoic data from ferromanganese crusts D11 and CD29 (in black) as published in Klemm et al.^27^ and Burton^28^, respectively, based on the updated age model of Nielsen et al.^29^. **c** Model estimates of the relative continental weathering feedback strength of the Cenozoic as published in Caves et al.^57^, based on their CO_2_ scenario 1 and a logarithmic expression for the weathering feedback

We present Re-Os data and Os_*i*_ values for middle Eocene sediments from Ocean Drilling Program (ODP) Site 959 in the equatorial Atlantic along the African continental margin, ODP Site 1263 on the Walvis Ridge in the South Atlantic, and Integrated Ocean Drilling Program (IODP) Site U1333 in the equatorial Pacific (Fig. 1; Supplementary Data 1; Supplementary Figs. 1–3). The Re and Os abundances are significantly enriched in the relatively organic-rich, siliceous sediments of Site 959 (Re = 10–60 ppb, Os = 100–300 ppt) relative to the carbonate-rich pelagic sediments of Sites 1263 and U1333 (Re = 0.02–0.2 ppb, Os = 10–40 ppt). The abundances of ^192^Os, the Os isotope best representing the amount of hydrogenous Os chelated by organic matter at the time of deposition^24,^ increase slightly over the study interval at Site 959, but are essentially stable at the other two sites (Fig. 1). We calculate Os_*i*_ values of 0.46 to 0.60 at all study sites (Fig. 1), which is in good agreement with previously published middle Eocene Os_*i*_ values from Site 959 sediments^25,26^ and with Os_*i*_ values from ferromanganese crusts that document a progressive increase in the ^187^Os/^188^Os composition of seawater during the Cenozoic^27–29^ (Fig. 2).

At Site 959, the Os_*i*_ values range between approximately 0.56 and 0.60 for most of the middle Eocene study interval, with the exception of a decrease to 0.51 during the MECO at ~580 mbsf (Fig. 1). Importantly, the lack of an increase in the Os_*i*_ values during the MECO implies that weathering rates of felsic silicate rocks did not increase in response to CO_2_ rise and accompanied warming, while such an increase would be expected from theory and published Os_*i*_ records from analogous carbon cycle perturbations^3,7,15^ (Fig. 2b). Furthermore, the relative invariability of both the Os_*i*_ records and the ^192^Os abundances—which scale to organic matter content—implies that the balance of Os fluxes to the oceans and uptake of Os in sedimentary organic matter did not appreciably change during the MECO.

Although the magnitude of the negative Os_*i*_ shift at Site 959 is small (~0.05), it exceeds the maximum analytical uncertainty (2*σ* = 0.01) by a factor of 5. The shift starts at the onset of MECO warming and is also present at Sites 1263 and U1333, where it is similar in magnitude (Figs. 1, 2). Interestingly, the Os_*i*_ profile of Site U1333 is characterized by two separate excursions to lower, less radiogenic values rather than the gradual and continuous decrease that is observed at Site 959. The Os_*i*_ profile at Site 1263 shows trends intermediate to Sites 959 and U1333. Nevertheless, the lowest Os_*i*_ values in all three records occur toward the end of the MECO, which is coincident with the peak warming phase^10^. In addition, the return towards pre-MECO values is synchronous with the termination of the MECO at all three sites, implying that the Os_*i*_ shift lasted for the entire duration of the event (~500 kyr). The absolute Os_*i*_ values differ slightly between sites, likely because of differences in coastal proximity and oceanographic setting^30,31^. However, the general timing and magnitude of the Os_*i*_ shift are reproduced at all sites, indicating that the Os_*i*_ shift records a change in the ^187^Os/^188^Os composition of the global ocean. The global character and synchroneity of the Os_*i*_ shift at the end of the MECO also indicate that osmium isotope stratigraphy is a promising tool for correlation of the event between sites in future studies (Fig. 2a).

In principle, the modest negative Os_*i*_ shift during the MECO may be caused by an increase in the unradiogenic Os flux from hydrothermal and/or extraterrestrial sources, a decrease in the radiogenic Os flux from continental weathering, or a decrease in the ^187^Os/^188^Os composition of the continental weathering flux through a transient change in the exposure of different rock types, such as basalts^7^. There is no evidence for an extraterrestrial impact during the MECO. Furthermore, a reduction in continental silicate weathering rates during an episode of greenhouse warming seems paradoxical and unlikely, even though our Os_*i*_ records clearly show no evidence of the expected increase in continental weathering. It is difficult to exclude a warming-induced change in regional climates and precipitation patterns—which could have affected the contributions of rock types with different ^187^Os/^188^Os compositions to the continental weathering flux^3,32^—as a cause for the Os_*i*_ shift. However, this would still require a different cause for MECO warming.

Finally, the Os_*i*_ shift could reflect a short-lived increase in mid-ocean ridge hydrothermal activity or an episode of increased volcanism and associated weathering of mafic silicate rocks^24,33,34^. Mass balance calculations with a progressive two-component mixing model that involves seawater and basalts (see Methods; Supplementary Data 2) show that the Os_*i*_ shift across the MECO may correspond to a 10–15% increase in the contribution of the mantle-derived Os flux relative to the continental Os flux. Although there is no indication for the emplacement of a large igneous province during the middle Eocene^8^, an episode of volcanic activity at mid-ocean ridges or on land could have increased the Os flux from basalts, and consequently resulted in a decrease of the ^187^Os/^188^Os composition of the oceans that is consistent with our Os_*i*_ records. Moreover, enhanced volcanism would provide a mechanism for the atmospheric CO_2_ rise that has been inferred for the MECO^8,11^, perhaps similar to the Late Cretaceous episode of greenhouse warming associated with volcanic eruptions from the Deccan Traps^33,35,36^. Potential events that have been dated at approximately the right age in the middle Eocene include (1) a pulse of metamorphic decarbonation associated with Himalayan uplift and metamorphism^37,38^, (2) increased arc volcanism around the Pacific rim^39^ and especially in the Caribbean, related to an ignimbrite flare-up in the Sierra Madre Occidental of Mexico^40–42^, (3) an episode of magmatism in the East African Rift zone^43^, in particular in Southern Ethiopia and Northern Kenya^44,45^, and/or (4) mid-ocean ridge volcanism in the North Atlantic, due to rifting in East Greenland and activity of the Iceland hotspot^46–48^. However, the timing and magnitude of these events are at present not sufficiently well resolved to establish a direct causal link with the MECO. Additionally, it is unclear if increased Himalayan uplift and metamorphism would be compatible with the observed negative Os_*i*_ shift, as the Himalayas are generally considered to contribute relatively radiogenic Os to the continental weathering flux^49,50^. Yet, the effects of Himalayan uplift and subsequent weathering on the Cenozoic Os_*i*_ record are likely small^51,52^.

### Carbon and osmium cycle modeling

Enhanced volcanism and/or hydrothermal activity may represent the most parsimonious scenario to explain the modest negative Os_*i*_ shift and atmospheric CO_2_ rise during the MECO. However, a strong silicate weathering response to greenhouse warming through focused weathering of fresh basalts is in disagreement with the extensive carbonate dissolution observed in deep ocean basins^8,10^. Therefore, total continental weathering fluxes must have remained approximately constant during the event. Collectively, the available data indicate that CO_2_ was added to the ocean-atmosphere system through enhanced volcanism, leading to warming, but was not neutralized through the silicate weathering feedback, leading to sustained ocean acidification.

**Fig. 3:**
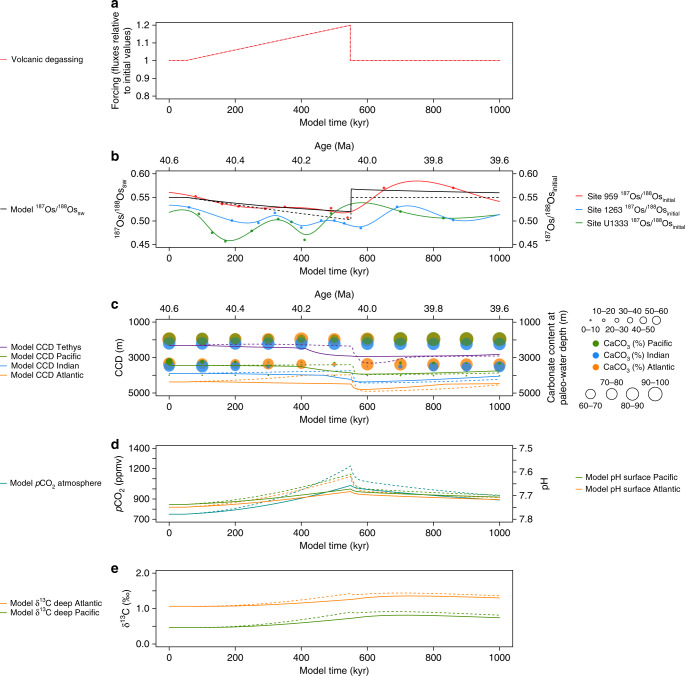
LOSCAR and Os cycle model simulations of the most likely MECO scenario. **a** Forcing for two scenarios involving a gradual, transient 20% increase in the volcanic CO_2_ flux over ~500 kyr. The solid lines represent a scenario in which the silicate and carbonate weathering fluxes are allowed to vary in response to CO_2_ forcing (normal weathering feedback), while the dashed lines represent a scenario in which these weathering fluxes are kept constant (diminished weathering feedback). Only the latter scenario corresponds to all observations. **b** Model response in the ^187^Os/^188^Os composition of the global ocean, shown against smoothed fits to the MECO Os_*i*_ records from the study sites. **c** Model CCD response of different ocean basins, shown against carbonate content (wt%) records for different depths in the Atlantic, Indian and Pacific oceans as compiled by Sluijs et al^8^. **d** Model atmospheric *p*CO_2_ response and pH response for the surface Atlantic and Pacific oceans. **e** Model δ^13^C response for the DIC of the deep Atlantic and Pacific oceans. For a full description of the LOSCAR model, see Zeebe^23^

To test the plausibility of scenarios involving enhanced volcanism and/or diminished continental weathering during the MECO, we performed a series of carbon cycle simulations with the box model LOSCAR^23^ by prescribing fluxes with the transient shift that is inferred from our Os_*i*_ records (see Methods; Fig. 3; Supplementary Figs. 4–9). For consistency, we have also modeled the ^187^Os/^188^Os composition of the global ocean by applying the same LOSCAR carbon cycle fluxes as forcing to a box model of the Os cycle (see Methods; Supplementary Software 1). In addition to a ~0.05 decrease in the ^187^Os/^188^Os ratio of seawater, our target scenario for the MECO involves a rise in atmospheric CO_2_ concentrations, a slight increase in the δ^13^C of dissolved inorganic carbon in the deep ocean, and a shoaling of the CCD over ~500 kyr^8^. Since there are no high-resolution *p*CO_2_ records available for the MECO, the target scenario includes an approximate doubling of atmospheric CO_2_ concentrations relative to middle Eocene background values of 500–1000 ppmv^11,53^. Furthermore, the magnitude of CCD change during the event possibly varied between the different ocean basins^10^, so we incorporate a conservative estimate of at least 500 m shoaling in the Atlantic and Pacific in our target scenario.

All model simulations result in a decrease in the ^187^Os/^188^Os ratio of seawater (Fig. 3; Supplementary Figs. 4–9). Although a gradual, linear increase in volcanism of 10–20% over ~500 kyr is sufficient to cause CO_2_ accumulation in the ocean-atmosphere system, and hence global warming on MECO timescales, this scenario results in a deepening of the CCD instead of the observed shoaling (Fig. 3; Supplementary Fig. 4)^8,10^. A similar behavior of the CCD is observed in previous LOSCAR simulations of the MECO^8^ and the Late Cretaceous warming episode^36^. Crucially, the model is only able to reproduce CO_2_ rise in conjunction with shoaling of the CCD on these timescales if we invoke enhanced volcanism together with a diminished weathering feedback by maintaining the silicate and carbonate weathering fluxes constant (Fig. 3; Supplementary Fig. 5). Although the magnitude of this modeled CCD response is smaller than the shoaling inferred from deep-sea carbonate records^8,10^, we note that the model may underestimate CCD changes because it does not account for the additional effects of biological carbonate compensation^54^. Such a reduction in net carbonate production resulting from ocean acidification might amplify the CCD response for a given CO_2_ rise. In all scenarios, the model reproduces the modest increase in deep-sea benthic foraminifer δ^13^C values during the event^9,10^ because of a resulting decrease in carbonate versus constant organic carbon burial. Finally, the relatively rapid termination of the MECO is forced by a recovery of silicate weathering. We note that this does not need to represent a pronounced tectonic event, as the absolute magnitude of the flux imbalances is relatively small, but regionally enhanced weathering in the aftermath of the MECO would be consistent with observations from the Tethys region^55^.

## Discussion

To reconcile our Os_*i*_ records and model results with global warming and atmospheric CO_2_ rise on MECO timescales^8–11^, we hypothesize that a long-term reduction in the strength of the negative silicate weathering feedback occurred in the Eocene^56,57^, due to a progressive reduction in the weatherability of the continents—the sum of all factors affecting chemical weathering other than climate^58,59^. For millions of years prior to the MECO, the Earth was generally characterized by high atmospheric CO_2_ levels^53^ and very warm climates^60^ (Supplementary Fig. 10), as well as flat continental relief. Thick, cation-depleted soils developed and transport-limited weathering regimes prevailed^61,62^, and consequently the weatherability of Earth’s surface may have gradually decreased over the course of the Eocene. Indeed, such a progressive reduction in weathering feedback strength during the Eocene has been inferred from inverse modeling simulations of weathering fluxes based on Cenozoic *p*CO_2_ and δ^13^C records^57^ (Fig. 2c). With the strength of the weathering feedback strongly diminished, a small increase in volcanism or hydrothermal activity would lead to the accumulation of large amounts of CO_2_ in the ocean-atmosphere system, resulting in prolonged warming and ocean acidification during the MECO.

Changes in weatherability have also been suggested to explain other episodes of apparent decoupling between silicate weathering and climate^59^, for example during major glaciations in the Paleozoic and Neoproterozoic^63–65^. Our interpretations of a limited weathering response during the MECO suggest that a variable silicate weathering feedback strength^57^ can indeed act as a driver for sustained global warming on geological timescales, with potential importance to other enigmatic phases of carbon cycle change in Earth’s history. Moreover, a variable weathering feedback strength governed by the interplay between tectonics, climate and the weatherability of the continents fundamentally challenges the parameterization of the silicate weathering feedback in carbon cycle models, especially those used to model transient perturbations such as the OAEs and the PETM. We therefore argue that future studies of these events should focus on exploring changes in temperature, atmospheric CO_2,_ and the CCD in conjunction with the strength of the weathering feedback.

## Methods

### Sampling

The samples used in this study were derived from middle Eocene sedimentary units from three different sites: organic-rich sediments from ODP Site 959 in the equatorial Atlantic along the African continental margin, carbonate-rich pelagic sediments from ODP Site 1263 on the Walvis Ridge in the south Atlantic, and carbonate-rich pelagic sediments from IODP Site U1333 in the equatorial Pacific (Supplementary Fig. 1). The total organic carbon (TOC) contents of these middle Eocene sediments range between 0.1 and 2%, with the highest TOC abundances occurring at Site 959^66^. Rock samples of 20–40 g were selected across the middle Eocene interval between approximately 42 and 38 Ma, with the highest sampling resolution across the MECO.

### Analyses

Bulk samples were freeze-dried or oven-dried at 50 °C and subsequently powdered using a ceramic pestle and mortar, in order to homogenize the Re and Os within the samples. Contact with metal surfaces was avoided so as not to contaminate the sample set. All Re and Os isotope analyses were performed at the Laboratory for Source Rock and Sulfide Geochronology and Geochemistry, and the Arthur Holmes Laboratory at the Durham Geochemistry Centre, Durham University (UK). Samples were digested in a CrO_3_-H_2_SO_4_ solution (0.25 g/g CrO_3_ in 8 mL of 4 N H_2_SO_4_) following the well-established methods of Selby & Creaser^67^, which have been shown to significantly limit the contribution of detrital Re and Os to the hydrogenous fraction bound to organic matter.

Powdered samples of approximately 0.3–1 g were loaded into Carius tubes with a known amount of ^185^Re + ^190^Os tracer solution (spike) and dissolved in 8 mL of CrO_3_-H_2_SO_4_ solution. The Carius tubes were then sealed and heated in an oven at 220 °C for 48 h. Osmium was isolated from the CrO_3_-H_2_SO_4_ sample solution by using solvent extraction with chloroform (CHCl_3_), and then back extracted by hydrobromic acid (HBr). The Os was further purified through micro distillation. Rhenium was isolated by evaporating 1 mL of the CrO_3_–H_2_SO_4_ sample solution to dryness, followed by solvent extraction involving sodium hydroxide (NaOH) and acetone (C_3_H_6_O)^68,69^. The Re was further purified by anion chromatography.

Following purification, the Re and Os fractions were loaded onto Ni and Pt filaments, respectively, together with 0.5 μL BaNO_3_ and BaOH activator solutions, respectively^67^. Rhenium and osmium isotope ratios were determined by negative thermal ionization mass spectrometry, using Faraday cups for Re and a Secondary Electron Multiplier for Os in peak-hopping mode.

Re and Os isotope ratios were corrected for instrumental mass fractionation, as well as spike and blank contributions. Procedural blanks for Re and Os in this study were 12 ± 3 pg/g and 0.07 ± 0.05 fg/g, respectively, with an ^187^Os/^188^Os value of 0.25 ± 0.15 (*n* = 3). The ^187^Re/^188^Os and ^187^Os/^188^Os uncertainties (2σ) include full propagation of uncertainties in weighing, mass spectrometer measurements, spike calibrations, blank corrections, and reproducibility of standards.

The ^187^Os/^188^Os_initial_ ratios (Os_*i*_) were calculated by correcting for post-depositional ^187^Re decay over time with the following equation:

1 $${}^{187}\mathrm{Os}/{}^{188}\mathrm{Os}_{{\mathrm{initial}}}\left( {\mathrm{Os}_{{i}}} \right) = {}^{187}\mathrm{Os}/{}^{188}\mathrm{Os}_{{\mathrm{measured}}} - {}^{187}\mathrm{Re}/{}^{188}\mathrm{Os}_{{\mathrm{measured}}} \ast \left( {{\mathrm{e}}^{\lambda t} - 1} \right)$$

where *λ* is the ^187^Re decay constant (1.666 · 10^−11^ yr^−1^)^70^ and *t* is the age of the rock. Given the high Re abundances in the organic-rich sediments from Site 959, we have used best estimates for the depositional ages of each of these samples. An age of 40 Ma was used for all samples from Sites 1263 and U1333, because improved age estimates would result in variations in Os_*i*_ values of 0.1% or less on average. All results are listed in Supplementary Data 1. The Re–Os isotopic system is expected to have remained closed for the sample set, given that the cores were all fresh, unweathered, and showed no evidence of post-depositional events (e.g., veining, etc.). Further, where the Re–Os data has sufficient spread in isochron plot space to yield statistically robust isochrons, a geologically reasonable Re–Os isochron age is obtained (e.g., Site 959; see below for details).

### Evaluation of Re and Os data

Although the studied samples were collected for evaluating changes in Os_*i*_ rather than establishing isochrons, the Re–Os data of the sediments from Site 959 show a positive correlation between ^187^Re/^187^Os and ^187^Os/^188^Os, which results in an isochron age that is in good agreement with the age of the MECO between 40.5 and 40.0 Ma (Supplementary Figs. 2, 3). In contrast, the ^187^Re/^187^Os and ^187^Os/^188^Os data for Sites 1263 and U1333 do not have sufficient spread in isochron plot space, and hence cannot yield statistically geologically meaningful age estimates.

### Age models

We adopt the age model of Cramwinckel et al.^13^ for Site 959 (Supplementary Fig. 11). This is based on initial^71^ and recently improved^13^ calcareous nannofossil biostratigraphy. The model also uses the long-term ^187^Os/^188^Os minimum at 34.65 Ma recorded at this site^26^, and TEX_86_ data that mark the MECO warming^13^. Moreover, we use the highest TEX_86_ value during the MECO peak warming and the lowest TEX_86_ value at the onset of the MECO as reported by Cramwinckel et al.^13^ to tentatively correlate to minima and maxima in the δ^18^O records of Bohaty et al.^10^, which were assigned ages of 40.06 and 40.52 Ma, respectively. Better age models are available for the other two sites. For Site 1263, we use a published age model^10^ based on magnetostratigraphy and bulk carbonate δ^18^O and δ^13^C chemostratigraphy. For Site U1333, an astronomically calibrated magnetostratigraphic age model^72^ was used in combination with calcareous nannofossil events^73^. All ages were adjusted to the framework of the GTS 2012^74^ and tie points for the age models are listed in Supplementary Tables 1, 2 and 3.

### Calculating changes in Os fluxes across the MECO

The ^187^Os/^188^Os composition of seawater is controlled by the balance between input fluxes from continental, mantle-derived, and extraterrestrial sources. However, the flux of extraterrestrial Os is generally assumed to be negligible and constant^75,76^, so our Os_*i*_ records can be used to directly infer changes in relative contributions of the continental and mantle-derived Os sources across the MECO. To evaluate an increase in the mantle-derived Os flux, we developed a progressive, two-component mixing model for the release of Os from mantle-derived basalts that incorporates both the Os abundance and ^187^Os/^188^Os composition of seawater and basalts. This model is an adaptation of the two-component mixing model for strontium (Sr) isotopes of Faure (1986, Equations (9.2) and (9.10))^77^, with modifications to consider the larger range of Os isotope variations in comparison to Sr isotope variations.

From the relative molar concentrations of natural Os isotopes, we know:

2 $$\frac{{\left[ {{\mathrm{Os}}} \right] - \left[ {\;{}^{187}{\mathrm{Os}}} \right]}}{{\left[ {\;{}^{188}{\mathrm{Os}}} \right]}} = 7.4$$

where [Os] represents the molar concentration (in mol/kg) of total Os (i.e., ^186^Os + ^187^Os + ^188^Os + ^189^Os + ^190^Os + ^192^Os), and [^187^Os] and [^188^Os] represent the molar concentrations (in mol/kg) of ^187^Os and ^188^Os, respectively^78^.

Equation (2) can be rewritten as:

3 $$\left[ {\;^{187}{\mathrm{Os}}} \right] = \frac{R}{{7.4 + R}}\left[ {{\mathrm{Os}}} \right]$$

4 $$\left[ {\;{}^{188}{\mathrm{Os}}} \right] = \frac{1}{{7.4 + R}}\left[ {{\mathrm{Os}}} \right]$$

where *R* = [^187^Os]/[^188^Os].

Two-component mixing between seawater and basalts can then be expressed for both ^187^Os and ^188^Os as:

5 $$\left[ {\;{}^{187}{\mathrm{Os}}} \right]_{{\mathrm{mix}}} = \frac{{\left[ {\;{}^{187}{\mathrm{Os}}} \right]_{{\mathrm{sw}}} \ast M_{{\mathrm{sw}}} + \left[ {\;{}^{187}{\mathrm{Os}}} \right]_{{\mathrm{bas}}} \ast M_{{\mathrm{bas}}}}}{{M_{{\mathrm{sw}}} + M_{{\mathrm{bas}}}}}$$

6 $$\left[ {\;{}^{188}{\mathrm{Os}}} \right]_{\mathrm{mix}} = \frac{{\left[ {\;{}^{188}{\mathrm{Os}}} \right]_{{\mathrm{sw}}} \ast M_{{\mathrm{sw}}} + \left[ {\;{}^{188}{\mathrm{Os}}} \right]_{{\mathrm{bas}}} \ast M_{{\mathrm{bas}}}}}{{M_{\mathrm{sw}} + M_{{\mathrm{bas}}}}}$$

where *M* represents the mass of a component (in kg) and the subscripts sw, bas and mix represent seawater, basalts and the eventual mix between the two, respectively.

We now define:

7 $$\Delta M_{{\mathrm{bas}}} = \frac{{M_{{\mathrm{bas}}}}}{{M_{{\mathrm{sw}},{\mathrm{initial}}}}}$$

8 $$f = \frac{{\Delta M_{{\mathrm{bas}}}}}{{M_{{\mathrm{sw}},{\mathrm{progressive}}} + \Delta M_{{\mathrm{bas}}}}} = \frac{{\Delta M_{{\mathrm{bas}}}}}{{M_{{\mathrm{mix}}}}}$$

where Δ*M*_bas_ is an infinitesimal representing the mass of basalts added during a mixing step relative to the mass of seawater initially present, and *f* represents the amount of basalts added during a mixing step relative to the total amount of seawater and basalts present during progressive mixing (*M*_mix_).

Equations (3)–(8) can then be combined as follows:

9 $$\begin{array}{l}\left[ {\;{}^{187}{\mathrm{Os}}} \right]_{{\mathrm{mix}}} = f \ast \left[ {\;{}^{187}{\mathrm{Os}}} \right]_{{\mathrm{bas}}} + \left( {1 - f} \right) \ast \left[ {\;{}^{187}{\mathrm{Os}}} \right]_{{\mathrm{sw}}}\\ \hskip 70pt= f \ast \frac{{R_{{\mathrm{bas}}}}}{{7.4 + R_{{\mathrm{bas}}}}}\left[ {{\mathrm{Os}}} \right]_{{\mathrm{bas}}} + \left( {1 - f} \right) \ast \frac{{R_{{\mathrm{sw}}}}}{{7.4 + R_{{\mathrm{sw}}}}}\left[ {{\mathrm{Os}}} \right]_{{\mathrm{sw}}}\end{array}$$

10 $$\begin{array}{l}\left[ {\;{}^{188}{\mathrm{Os}}} \right]_{{\mathrm{mix}}} = f \ast \left[ {\;{}^{188}{\mathrm{Os}}} \right]_{{\mathrm{bas}}} + \left( {1 - f} \right) \ast \left[ {\;{}^{188}{\mathrm{Os}}} \right]_{{\mathrm{sw}}}\\ \hskip 70pt= f \ast \frac{1}{{7.4 + R_{{\mathrm{bas}}}}}\left[ {{\mathrm{Os}}} \right]_{{\mathrm{bas}}} + \left( {1 - f} \right) \ast \frac{1}{{7.4 + R_{{\mathrm{sw}}}}}\left[ {{\mathrm{Os}}} \right]_{{\mathrm{sw}}}\end{array}$$

Finally, dividing equation (9) by equation (10) yields:

11 $$\begin{array}{l}R_{{\mathrm{mix}}} = \frac{{\left[ {\;{}^{187}{\mathrm{Os}}} \right]_{{\mathrm{mix}}}}}{{\left[ {\;{}^{188}{\mathrm{Os}}} \right]_{{\mathrm{mix}}}}} = \frac{{f \ast \frac{{R_{{\mathrm{bas}}}}}{{7.4 + R_{{\mathrm{bas}}}}}\left[ {{\mathrm{Os}}} \right]_{{\mathrm{bas}}} + \left( {1 - f} \right) \ast \frac{{R_{{\mathrm{sw}}}}}{{7.4 + R_{{\mathrm{sw}}}}}\left[ {{\mathrm{Os}}} \right]_{{\mathrm{sw}}}}}{{f \ast \frac{1}{{7.4 + R_{{\mathrm{bas}}}}}\left[ {{\mathrm{Os}}} \right]_{{\mathrm{bas}}} + \left( {1 - f} \right) \ast \frac{1}{{7.4 + R_{{\mathrm{sw}}}}}\left[ {{\mathrm{Os}}} \right]_{{\mathrm{sw}}}}}\end{array}$$

where *R* is the ^187^Os/^188^Os composition of the corresponding components (i.e., seawater, basalts, and the eventual mix between the two). Equations (7)–(11) can then be used to estimate the extent of mixing between seawater and basalts during the MECO by progressively calculating *R*_mix_ until our observed Os_*i*_ shift is reproduced (see Supplementary Data 2). We assumed the pre-MECO ^187^Os/^188^Os ratio of seawater to be ~0.55 based on an average of pre-MECO Os_*i*_ values recorded for the three sites and the Os concentration of seawater to be 10 ppq (~53 fmol/kg, similar to present-day values)^14^. Furthermore, we used an ^187^Os/^188^Os ratio of 0.13 for the mantle and mantle-derived basalts^79,80^, as well as an Os abundance of 1 ppt (~5.3 pmol/kg) for basalts^80^. Finally, we assumed that the maximum amount of basalt that can theoretically be added to seawater represents ~1% of the total mass of the ocean, as estimated for OAE2^31,81^, and used increments of 0.01% for the value of Δ*M*_bas_.

Based on an Os_*i*_ shift of 0.05 from the pre-MECO value of ~0.55 to a peak MECO value of ~0.50, we calculated a relative increase in the mantle-derived Os flux of ~13% across the event, which would equal the addition of Os from basalts with a mass of ~0.13% relative to the total mass of the ocean (Supplementary Data 2). Similar results are obtained if we estimate the relative increase in the ^188^Os flux, rather than the total Os flux. It is important to note that mantle-derived Os could also have been released to seawater through direct addition from magmatic degassing or hydrothermal inputs instead of basalt dissolution, but regardless of the mechanism, a ~13% increase in the mantle-derived Os flux during the MECO would be sufficient to reproduce our observed Os_*i*_ shift and would correspond to the cumulative release of ~9.4 · 10^6^ mol of mantle-derived Os. We also performed our calculations with the Os_*i*_ values of each individual site: for Site 959, an Os_*i*_ shift from 0.560 to 0.505 would yield a relative increase in the mantle-derived Os flux of ~14%; for Site 1263, an Os_*i*_ shift from 0.530 to 0.485 would yield an increase of ~12%; for Site U1333, an Os_*i*_ shift from 0.515 to 0.460 would yield an increase of ~16%. These differences are most likely to be attributed to the resolution of our records. To accommodate for this range of flux estimates, we adopted a best estimate of 10–15% for the increase in the mantle-derived Os flux during the MECO, but also explored the effects of an increase of up to 20% because we are unlikely to have sampled the lowest Os_*i*_ values in any of our records due to the relatively low resolution of our dataset.

### LOSCAR and Os cycle modeling

Carbon cycle simulations were performed using the Long-term Ocean-atmosphere-Sediment CArbon cycle Reservoir (LOSCAR) model^23^. In this box model, modified from Walker and Kasting^82^, carbon and several other biogeochemical tracers (e.g., alkalinity, phosphate, oxygen) are cycled through atmospheric and oceanic reservoirs. The model ocean is coupled to a sediment module and consists of surface-water, intermediate-water, and deep-water boxes of the four main Paleogene ocean basins (Atlantic, Indian, Pacific and Tethys). The model is designed to simulate the PETM at 56 Ma, but the minor changes in paleogeography compared to the middle Eocene at 40 Ma are not of relevance to the simple LOSCAR model. In these simulations, we use default parameter settings for the Paleogene setup. Equilibrium *p*CO_2_ is set at 750 ppm, consistent with *p*CO_2_ estimates based on planktic foraminifer boron isotope ratios (δ^11^B)^53^, and by default, silicate and carbonate weathering are implemented in the model as a feedback response to atmospheric CO_2_ concentrations. The CCD definition follows the default LOSCAR setup and is taken as the sediment depth level at which sedimentary CaCO_3_ contents fall below 10 wt%.

We explored the effects of changes in volcanism and/or continental weathering with the constraints from our Os_*i*_ records to assess which scenario is able to reproduce a more realistic MECO target. We first simulated several scenarios with a gradual, linear increase in the volcanic CO_2_ flux (+10, +15, and +20%) over ~500 kyr, either while allowing the silicate and carbonate weathering fluxes to vary in response to CO_2_ forcing (Supplementary Fig. 4), or while maintaining these weathering fluxes at constant values (Supplementary Fig. 5). Subsequently, we performed several simulations invoking silicate weathering as a forcing rather than a feedback, by prescribing a gradual, linear decrease in the silicate weathering flux (−10, −15, and −20%) over ~500 kyr, while keeping the volcanic CO_2_ flux and the carbonate weathering flux at constant values (Supplementary Fig. 6). Finally, we tested the effect of an increase in volcanism (+5%) combined with a decrease in silicate weathering (−5%) (Supplementary Fig. 7); the effect of a combined decrease in silicate and carbonate weathering (both −10%) (Supplementary Fig. 8); and the effect of a decrease in silicate weathering (−10%) while maintaining a carbonate weathering feedback (Supplementary Fig. 9). For an overview of all model scenarios, see Supplementary Table 4.

In order to demonstrate that our LOSCAR model simulations are consistent with the Os_*i*_ records, the scenarios outlined above were also applied to a separate box model of the Os cycle. This Os cycle model is inspired by the work of Richter & Turekian^83^ and many subsequent studies, including Peucker-Ehrenbrink & Ravizza^14^. We fully derive the equations used to model the Os cycle in the ocean below.

We first define *N* as the total molar inventory of Os (including all Os isotopes) in seawater, and ^187^*N* and ^188^*N* as the molar inventories of ^187^Os and ^188^Os in seawater, respectively. The ^187^Os/^188^Os composition of seawater (*R*_sw_) is thus expressed as:

12 $$R_{{\mathrm{sw}}} = \frac{{\;{}^{187}N}}{{\;{}^{188}N}}$$

Subsequently, changes in *R*_sw_ over time can be written as:

13 $$\begin{array}{l}\frac{{{\mathrm{d}}R_{{\mathrm{sw}}}}}{{{\mathrm{d}}t}} = \frac{{\mathrm{d}}}{{{\mathrm{d}}t}}\left( {\frac{{\,^{187}N}}{{\,^{188}N}}} \right) = \frac{{\,^{188}N\frac{{{\mathrm{d}}^{187}N}}{{{\mathrm{d}}t}} \,- \,^{187}N\frac{{{\mathrm{d}}^{188}N}}{{{\mathrm{d}}t}}}}{{\left( {\,^{188}N} \right)^2}} = \frac{1}{{\,^{188}N}}\left[ {\frac{{{\mathrm{d}}^{187}N}}{{{\mathrm{d}}t}} - R_{{\mathrm{sw}}}\frac{{{\mathrm{d}}^{188}N}}{{{\mathrm{d}}t}}} \right]\end{array}$$

Multiplying equation (13) by ^188^*N* gives:

14 $$\;{}^{188}N\frac{{{\mathrm{d}}R_{{\mathrm{sw}}}}}{{{\mathrm{d}}t}} = \frac{{{\mathrm{d}}{}^{187}N}}{{{\mathrm{d}}t}} - R_{{\mathrm{sw}}}\frac{{{\mathrm{d}}{}^{188}N}}{{{\mathrm{d}}t}}$$

Changes in *N*, ^187^*N* and ^188^*N* over time can then be written as follows:

15 $$\frac{{{\mathrm{d}}N}}{{{\mathrm{d}}t}} = F_{{\mathrm{riv}}} + F_{{\mathrm{hyd}}} + F_{{\mathrm{ext}}} - F_{{\mathrm{sed}}}$$

16 $$\begin{array}{l}\frac{{{\mathrm{d}}{}^{187}N}}{{{\mathrm{d}}t}} = F_{{\mathrm{riv}}}\left( {\frac{{\left[ {\;{}^{187}{\mathrm{Os}}} \right]}}{{\left[ {{\mathrm{Os}}} \right]}}} \right)_{{\mathrm{riv}}} + \,F_{{\mathrm{hyd}}}\left( {\frac{{\left[ {\;{}^{187}{\mathrm{Os}}} \right]}}{{\left[ {{\mathrm{Os}}} \right]}}} \right)_{{\mathrm{hyd}}}+ \,F_{{\mathrm{ext}}}\left( {\frac{{\left[ {\;{}^{187}{\mathrm{Os}}} \right]}}{{\left[ {{\mathrm{Os}}} \right]}}} \right)_{{\mathrm{ext}}} - \,F_{{\mathrm{sed}}}\left( {\frac{{\left[ {\;{}^{187}{\mathrm{Os}}} \right]}}{{\left[ {{\mathrm{Os}}} \right]}}} \right)_{{\mathrm{sed}}}\end{array}$$

17 $$\begin{array}{l}\frac{{{\mathrm{d}}{}^{188}N}}{{{\mathrm{d}}t}} = F_{{\mathrm{riv}}}\left( {\frac{{\left[ {\;{}^{188}{\mathrm{Os}}} \right]}}{{\left[ {{\mathrm{Os}}} \right]}}} \right)_{{\mathrm{riv}}} + \,F_{{\mathrm{hyd}}}\left( {\frac{{\left[ {\;{}^{188}{\mathrm{Os}}} \right]}}{{\left[ {{\mathrm{Os}}} \right]}}} \right)_{{\mathrm{hyd}}}\,+ \,F_{{\mathrm{ext}}}\left( {\frac{{\left[ {\;{}^{188}{\mathrm{Os}}} \right]}}{{\left[ {{\mathrm{Os}}} \right]}}} \right)_{{\mathrm{ext}}} - \,F_{{\mathrm{sed}}}\left( {\frac{{\left[ {\;{}^{188}{\mathrm{Os}}} \right]}}{{\left[ {{\mathrm{Os}}} \right]}}} \right)_{{\mathrm{sed}}}\end{array}$$

where *F* represents the fluxes of Os (in mol/yr) from and to various reservoirs and the subscripts sw, riv, hyd, ext and sed represent seawater, riverine, hydrothermal, extraterrestrial and sediment reservoirs, respectively^14,83^.

Substituting equations (3) and (4) into equations (16) and (17), respectively, yields:

18 $$\begin{array}{l}\frac{{{\mathrm{d}}{}^{187}N}}{{{\mathrm{d}}t}} = F_{{\mathrm{riv}}}\frac{{R_{{\mathrm{riv}}}}}{{7.4 + R_{{\mathrm{riv}}}}} + F_{{\mathrm{hyd}}}\frac{{R_{{\mathrm{hyd}}}}}{{7.4 + R_{{\mathrm{hyd}}}}}+\, F_{{\mathrm{ext}}}\frac{{R_{{\mathrm{ext}}}}}{{7.4 + R_{{\mathrm{ext}}}}} - F_{{\mathrm{sed}}}\frac{{R_{{\mathrm{sed}}}}}{{7.4 + R_{{\mathrm{sed}}}}}\end{array}$$

19 $$\begin{array}{l}\frac{{{\mathrm{d}}{}^{188}N}}{{{\mathrm{d}}t}} = F_{{\mathrm{riv}}}\frac{1}{{7.4 + R_{{\mathrm{riv}}}}} + F_{{\mathrm{hyd}}}\frac{1}{{7.4 + R_{{\mathrm{hyd}}}}}+ F_{{\mathrm{ext}}}\frac{1}{{7.4 + R_{{\mathrm{ext}}}}} - F_{{\mathrm{sed}}}\frac{1}{{7.4 + R_{{\mathrm{sed}}}}}\end{array}$$

Finally, substituting equations (18) and (19) into equation (14) and combining with equation (4) results in:

20 $$\begin{array}{l}\frac{N}{{7.4 + R_{{\mathrm{sw}}}}}\frac{{{\mathrm{d}}R_{{\mathrm{sw}}}}}{{{\mathrm{d}}t}} = F_{{\mathrm{riv}}}\frac{{R_{{\mathrm{riv}}} - R_{{\mathrm{sw}}}}}{{7.4 + R_{{\mathrm{riv}}}}} + F_{{\mathrm{hyd}}}\frac{{R_{{\mathrm{hyd}}} - R_{{\mathrm{sw}}}}}{{7.4 + R_{{\mathrm{hyd}}}}}+ F_{{\mathrm{ext}}}\frac{{R_{{\mathrm{ext}}} - R_{{\mathrm{sw}}}}}{{7.4 + R_{{\mathrm{ext}}}}} - F_{{\mathrm{sed}}}\frac{{R_{{\mathrm{sed}}} - R_{{\mathrm{sw}}}}}{{7.4 + R_{{\mathrm{sed}}}}}\end{array}$$

which relates changes in *R*_sw_ over time to the fluxes of total Os (*F*), the ^187^Os/^188^Os compositions of these fluxes (*R*) and the amount of total Os in the ocean (*N*). Because there is no isotopic fractionation associated with Os burial (i.e., *R*_sed_ = *R*_sw_), the net effect of the sedimentary Os flux (*F*_sed_) in equation (20) is zero.

Together, equations (15) and (20) can be used to simulate any transient perturbation of the Os cycle. We first constructed a steady state model based on flux estimates and ^187^Os/^188^Os values for the present-day Os cycle with a ^187^Os/^188^Os ratio of seawater of 1.06 (see Supplementary Table 5). For the middle Eocene Os cycle, we assumed that the total Os inventory and the total input and output fluxes of Os are similar to present-day values, and recalculated the steady state riverine and hydrothermal Os fluxes for the pre-MECO ^187^Os/^188^Os ratio of seawater of 0.55 by assuming that the ^187^Os/^188^Os composition of these fluxes has remained unchanged. Subsequently, we used scaled silicate weathering and volcanic degassing fluxes from the LOSCAR model simulations to force our model of the Os cycle. The modeled changes in the ^187^Os/^188^Os ratio of seawater are included in the respective figures of all model scenarios (Fig. 3 of the main text and Supplementary Figs. 4–9). The full code used to perform the Os cycle model simulations is included as an R script in Supplementary Software 1.

### Data availability

The authors declare that all data supporting the results of this study are available in the Supplementary Information files associated with this manuscript.

## Electronic supplementary material

Supplementary Information

Description of Additional Supplementary Files

Supplementary Data 1

Supplementary Data 2

Supplementary Software 1
