# Supplementary material for: Middle Eocene greenhouse warming facilitated by diminished weathering feedback
Source: Nat Commun. 2018 Jul 23;9:2877. doi: 10.1038/s41467-018-05104-9 (PMC6056486; doi:10.1038/s41467-018-05104-9)
Supplement: Supplementary file 1 — Supplementary Information [file 41467_2018_5104_MOESM1_ESM.pdf]

Middle Eocene greenhouse warming facilitated by diminished weathering feedback

Van der Ploeg et al.

Supplementary Information

This PDF file includes:

- Supplementary Figures 1 – 11
- Supplementary Tables 1 – 5
- Supplementary References 1 – 18

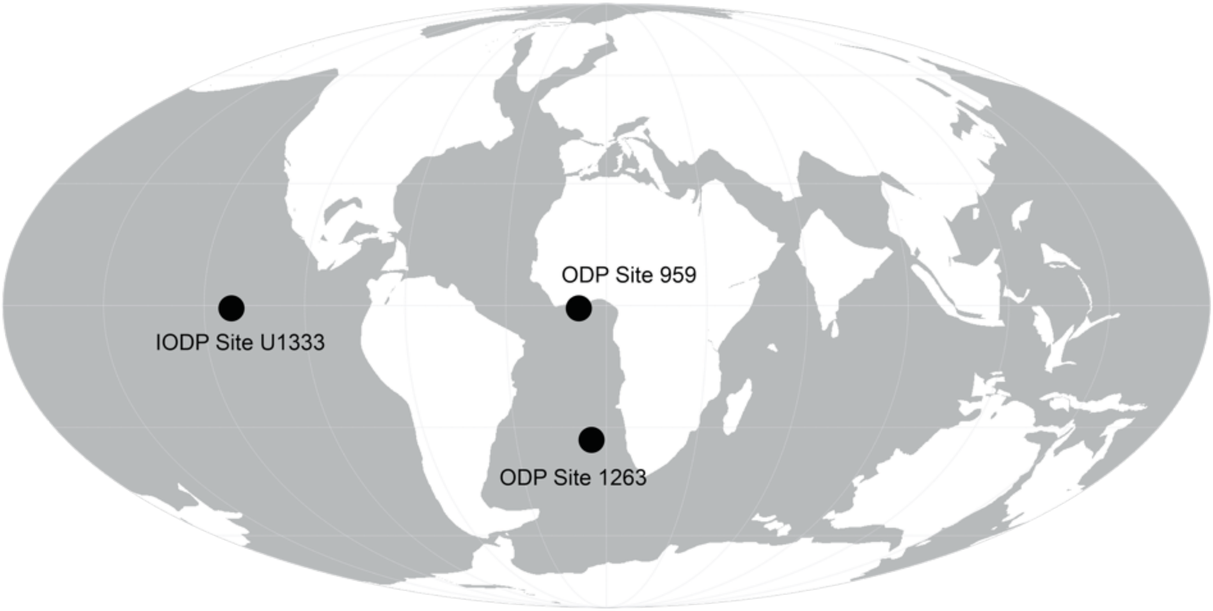

Supplementary Figure 1: Paleogeographic reconstruction of 40 Ma showing the estimated locations of the study sites. Shown are ODP Site 959 in the equatorial Atlantic along the African continent, ODP Site 1263 on the Walvis Ridge in the south Atlantic and IODP Site U1333 in the equatorial Pacific. The map was made with GPlates, based on the tectonic reconstructions of Seton et al. (2012)[1] and the paleomagnetic reference frame of Torsvik et al. (2012)[2].

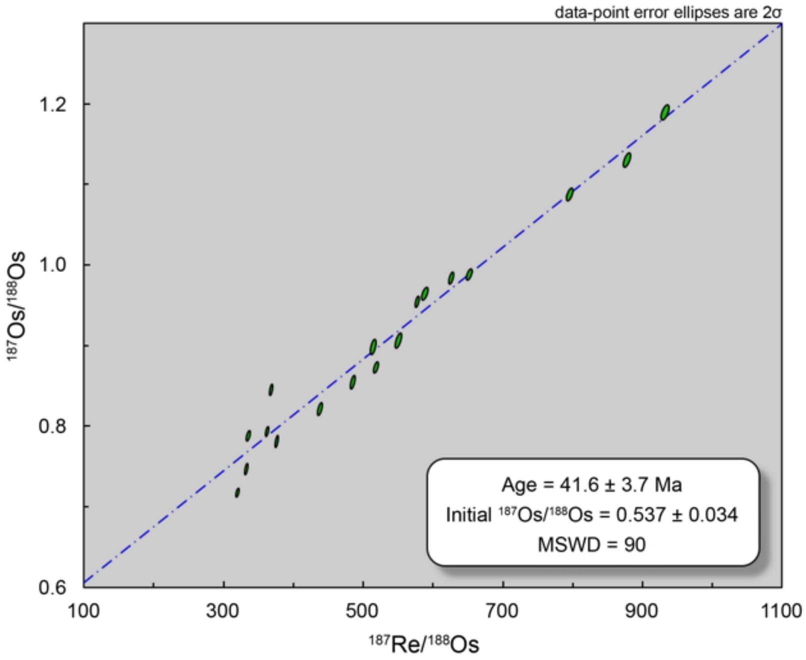

Supplementary Figure 2: Re-Os isochron plot of all Site 959 samples. The significant scatter (MSWD = 90) is best explained by the sample set possessing slightly variable initial $^{187}Os/^{188}Os$ compositions and being deposited over a prolonged interval of time (i.e., several Myr).

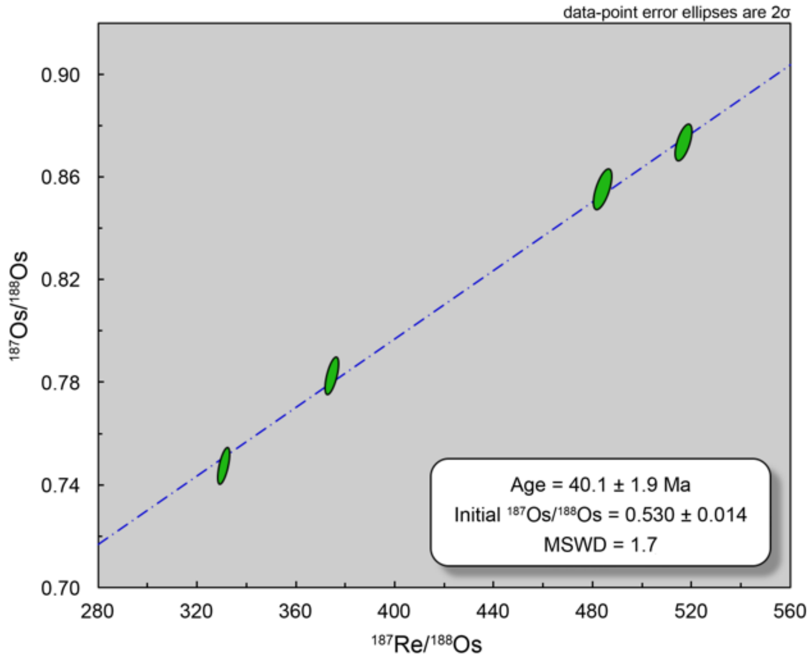

Supplementary Figure 3: Re-Os isochron plot of Site 959 samples in the MECO interval between 600.35 and 581.51 mbsf. These samples were selected because they were deposited in a short time interval (i.e., ~500 kyr), and yield virtually identical initial $^{187}Os/^{188}Os$ compositions. The obtained isochron age of 40.1 Ma is in excellent agreement with the estimated ages of these samples – between 40.4 and 40.1 Ma – based on our age model for Site 959.

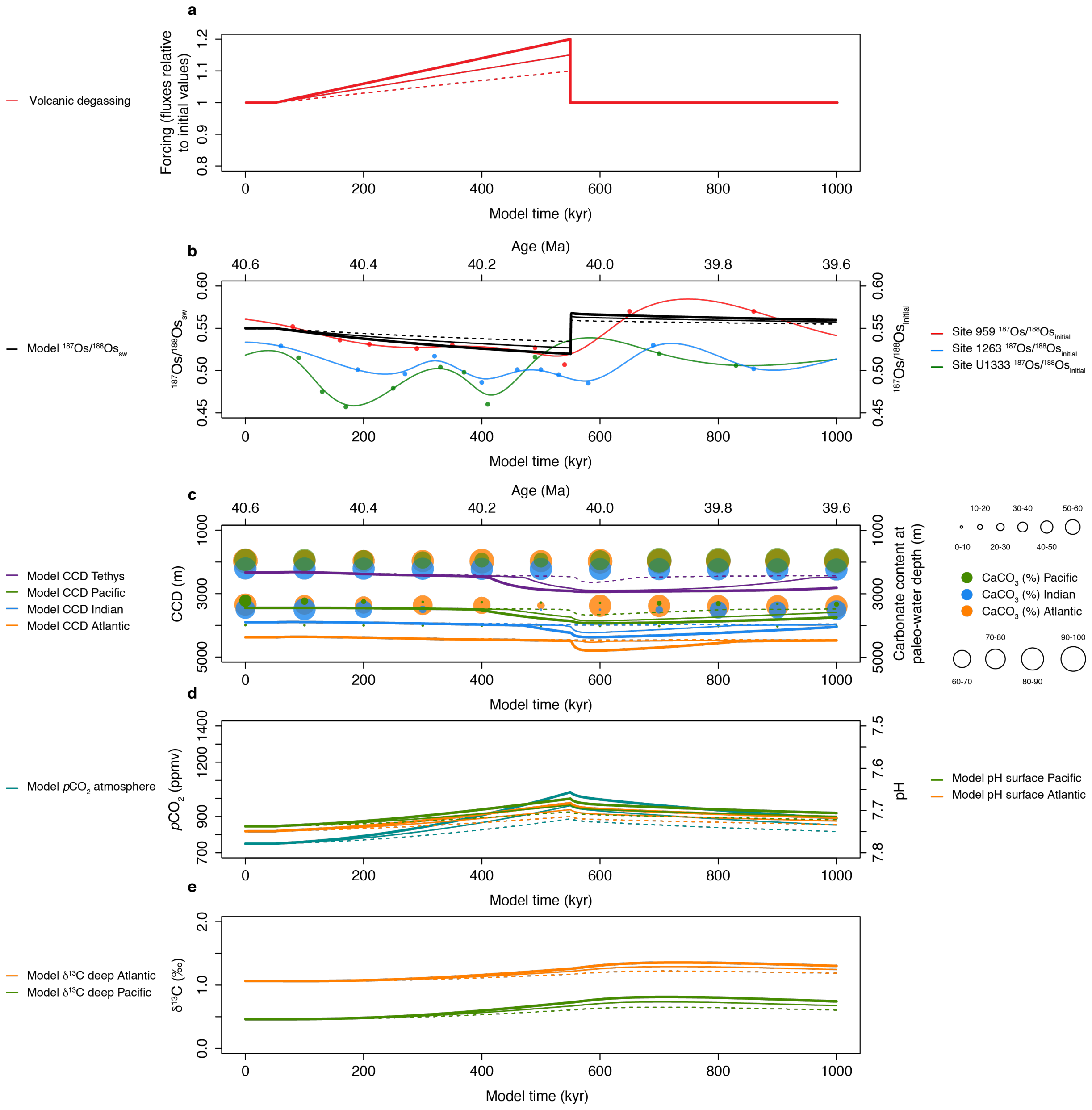

Supplementary Figure 4: LOSCAR and Os cycle model simulations of the MECO. a, Forcing for three scenarios involving a transient increase in the volcanic CO_2 flux of 10% (dashed lines), 15% (thin solid lines) and 20% (thick solid lines) over ~500 kyr, while allowing the silicate and carbonate weathering fluxes to vary as a feedback response. **b,** Model response in the $^{187}Os/^{188}Os$ composition of the global ocean, shown against smoothed fits to the MECO Os_i records from the study sites. **c,** Model CCD response of different ocean basins, shown against carbonate content (wt %) records for different depths in the Atlantic, Indian and Pacific oceans as compiled by Sluijs et al. (2013)[3]. **d,** Model atmospheric pCO_2 response and pH response for the surface Atlantic and Pacific oceans. **e,** Model $\delta^{13}C$ response for the DIC of the deep Atlantic and Pacific oceans.

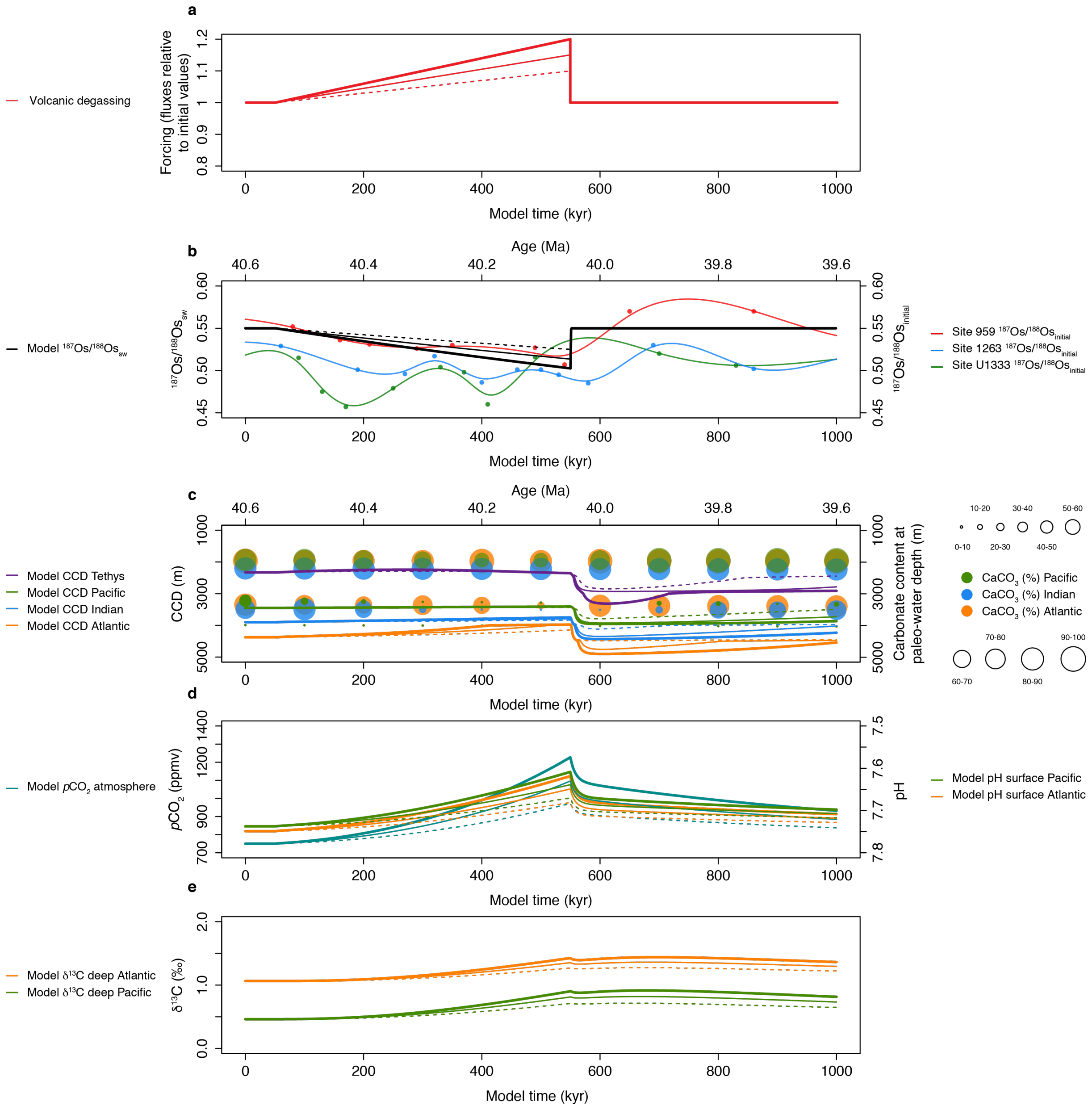

Supplementary Figure 5: LOSCAR and Os cycle model simulations of the MECO. a, Forcing for three scenarios involving a transient increase in the volcanic CO_2 flux of 10% (dashed lines), 15% (thin solid lines) and 20% (thick solid lines) over ~500 kyr, while maintaining the silicate and carbonate weathering fluxes at constant value. **b,** Model response in the $^{187}Os/^{188}Os$ composition of the global ocean, shown against smoothed fits to the MECO Os_i records from the study sites. **c,** Model CCD response of different ocean basins, shown against carbonate content (wt %) records for different depths in the Atlantic, Indian and Pacific oceans as compiled by Sluijs et al. (2013)[3]. **d,** Model atmospheric pCO_2 response and pH response for the surface Atlantic and Pacific oceans. **e,** Model $\delta^{13}C$ response for the DIC of the deep Atlantic and Pacific oceans.

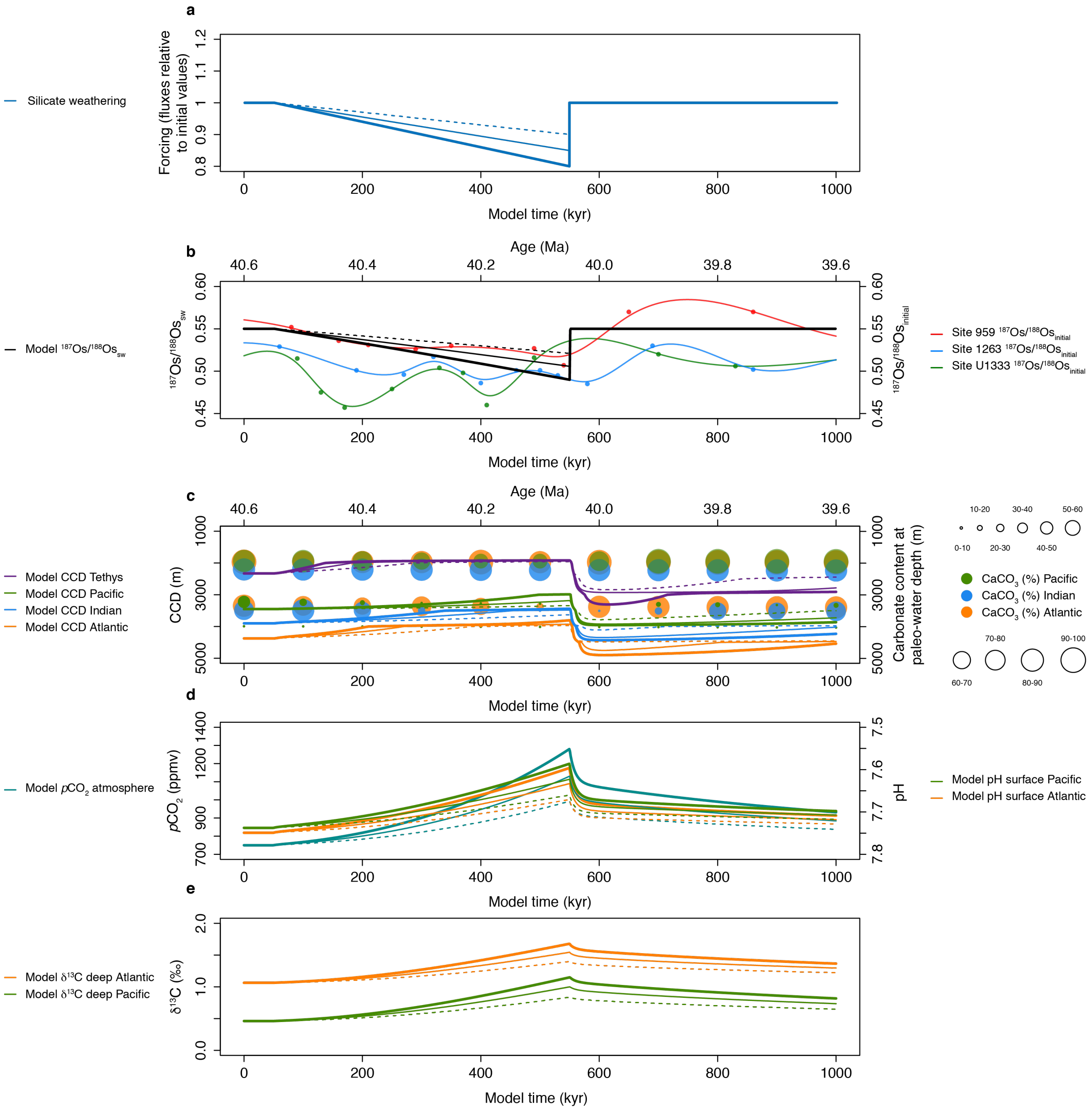

Supplementary Figure 6: LOSCAR and Os cycle model simulations of the MECO. a, Forcing for three scenarios involving a transient decrease in the silicate weathering flux of 10% (dashed lines), 15% (thin solid lines) and 20% (thick solid lines) over ~500 kyr, while keeping the volcanic CO_2 flux and the carbonate weathering flux at constant value. **b,** Model response in the $^{187}Os/^{188}Os$ composition of the global ocean, shown against smoothed fits to the MECO Os_i records from the study sites. **c,** Model CCD response of different ocean basins, shown against carbonate content (wt %) records for different depths in the Atlantic, Indian and Pacific oceans as compiled by Sluijs et al. (2013)[3]. **d,** Model atmospheric pCO_2 response and pH response for the surface Atlantic and Pacific oceans. **e,** Model $\delta^{13}C$ response for the DIC of the deep Atlantic and Pacific oceans.

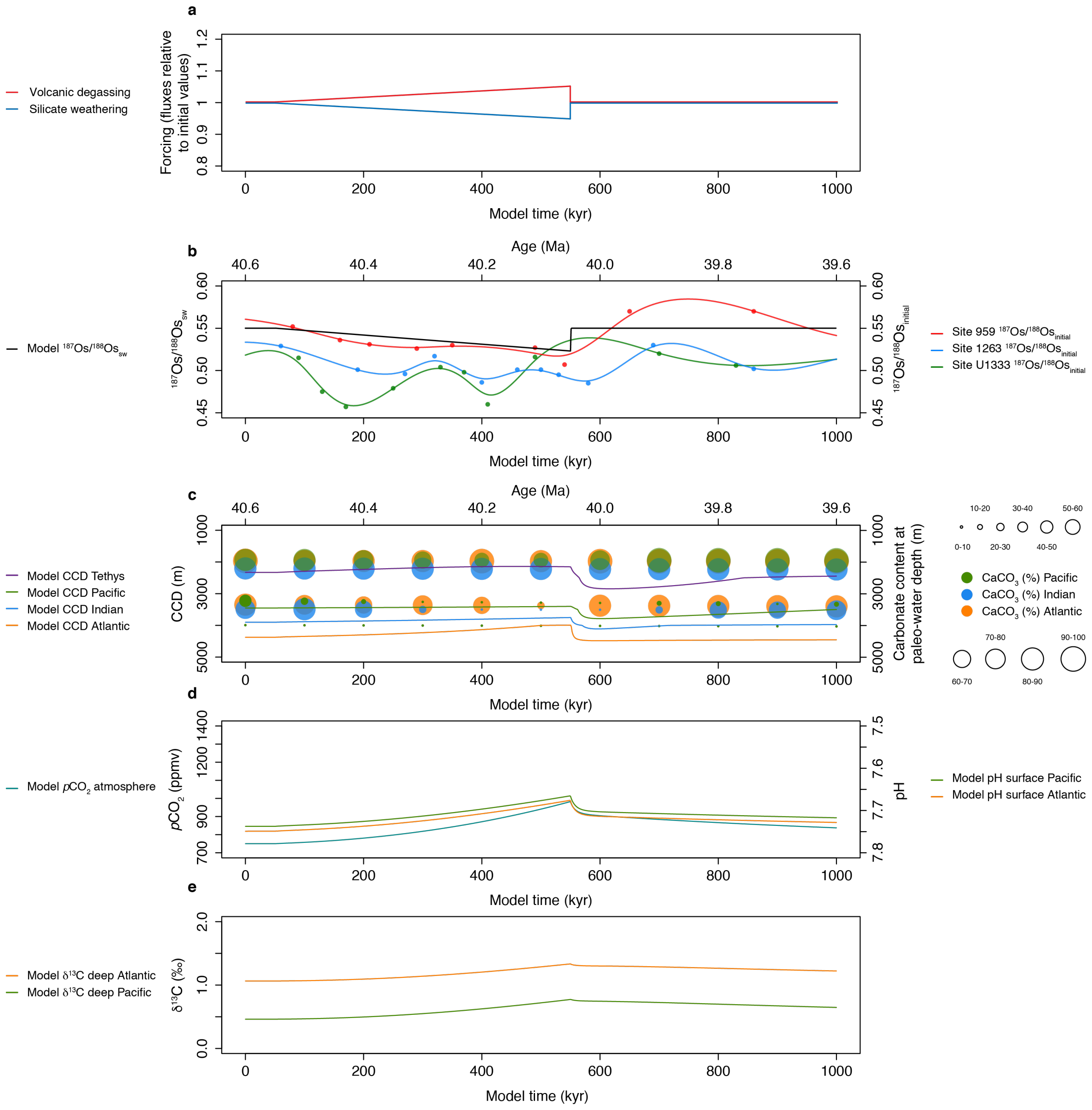

Supplementary Figure 7: LOSCAR and Os cycle model simulations of the MECO. a, Forcing for a scenario involving a transient 5% increase in the volcanic CO_2 flux combined with a 5% decrease in the silicate weathering flux over ~500 kyr, while keeping the carbonate weathering flux at constant value. **b,** Model response in the $^{187}Os/^{188}Os$ composition of the global ocean, shown against smoothed fits to the MECO Os_i records from the study sites. **c,** Model CCD response of different ocean basins, shown against carbonate content (wt %) records for different depths in the Atlantic, Indian and Pacific oceans as compiled by Sluijs et al. (2013)[3]. **d,** Model atmospheric pCO_2 response and pH response for the surface Atlantic and Pacific oceans. **e,** Model $\delta^{13}C$ response for the DIC of the deep Atlantic and Pacific oceans.

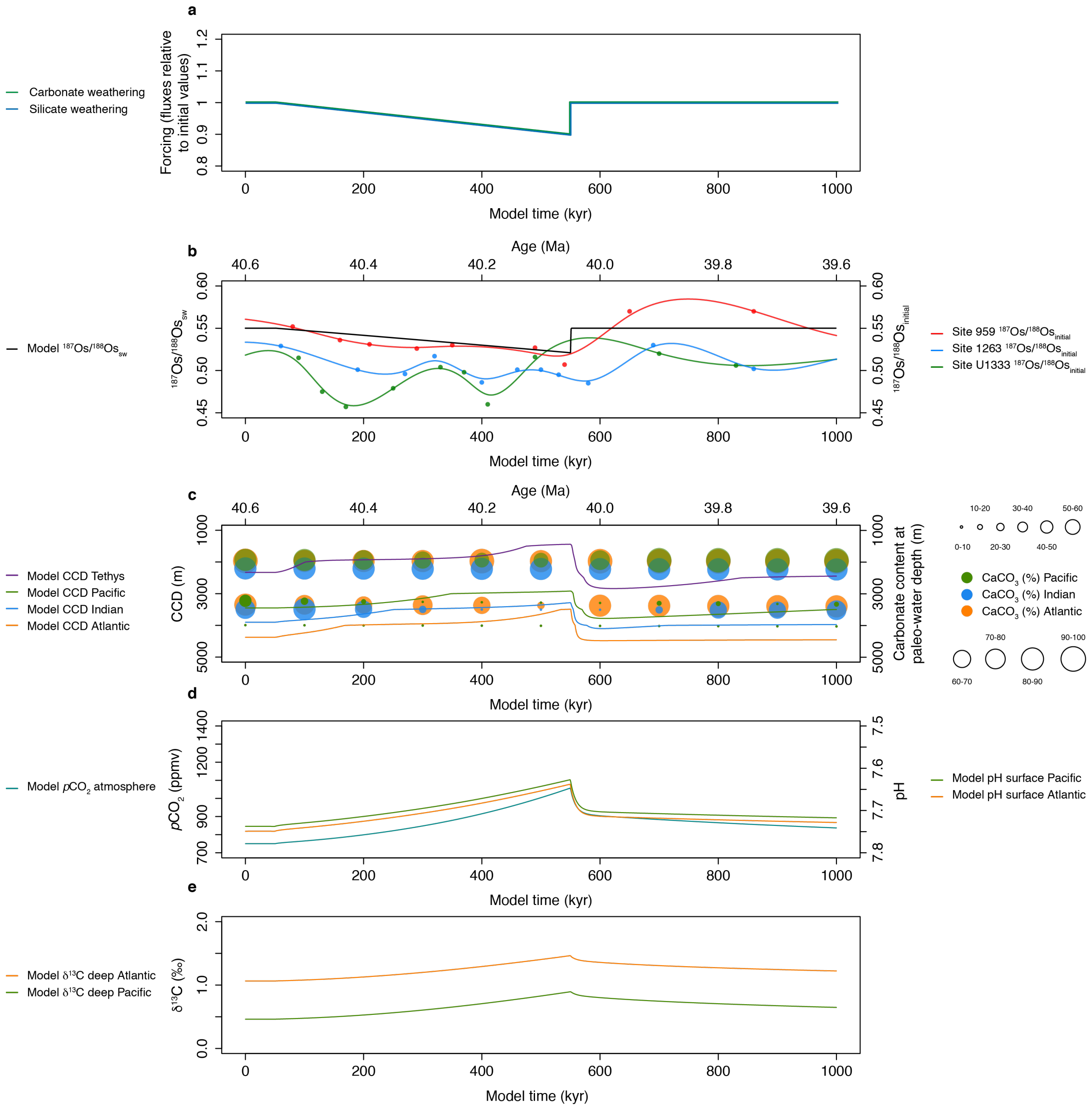

Supplementary Figure 8: LOSCAR and Os cycle model simulations of the MECO. a, Forcing for a scenario involving a transient, combined 10% decrease in the silicate and carbonate weathering fluxes over ~500 kyr, while keeping the volcanic CO_2 flux at constant value. **b,** Model response in the $^{187}Os/^{188}Os$ composition of the global ocean, shown against smoothed fits to the MECO Os_i records from the study sites. **c,** Model CCD response of different ocean basins, shown against carbonate content (wt %) records for different depths in the Atlantic, Indian and Pacific oceans as compiled by Sluijs et al. (2013)[3]. **d,** Model atmospheric pCO_2 response and pH response for the surface Atlantic and Pacific oceans. **e,** Model $\delta^{13}C$ response for the DIC of the deep Atlantic and Pacific oceans.

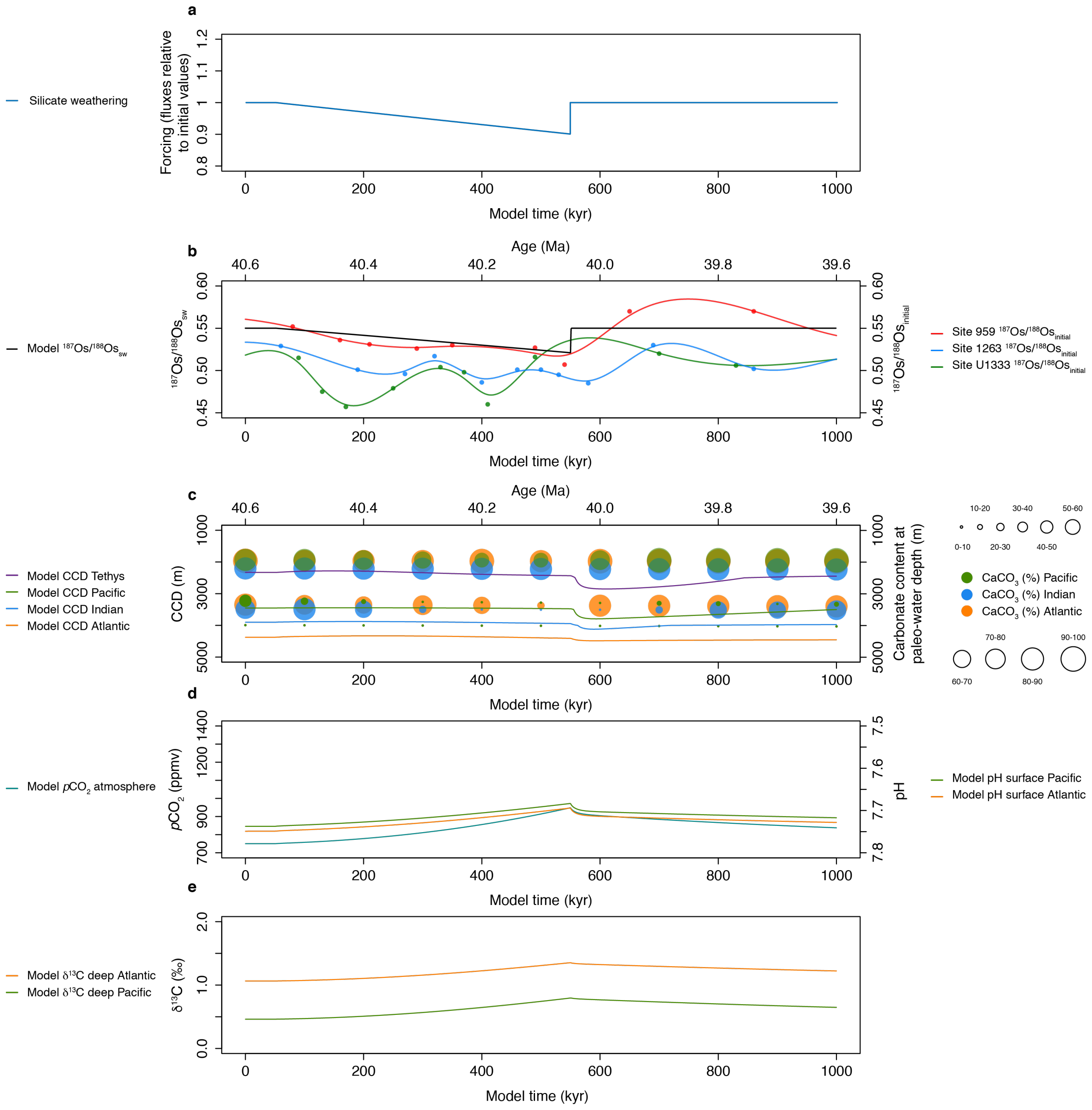

Supplementary Figure 9: LOSCAR and Os cycle model simulations of the MECO. a, Forcing for a scenario involving a transient 10% decrease in the silicate weathering flux over ~500 kyr, while keeping the volcanic CO_2 flux at constant value and allowing the carbonate weathering flux to vary as a feedback response. **b,** Model response in the $^{187}Os/^{188}Os$ composition of the global ocean, shown against smoothed fits to the MECO Os_i records from the study sites. **c,** Model CCD response of different ocean basins, shown against carbonate content (wt %) records for different depths in the Atlantic, Indian and Pacific oceans as compiled by Sluijs et al. (2013)[3]. **d,** Model atmospheric pCO_2 response and pH response for the surface Atlantic and Pacific oceans. **e,** Model $\delta^{13}C$ response for the DIC of the deep Atlantic and Pacific oceans.

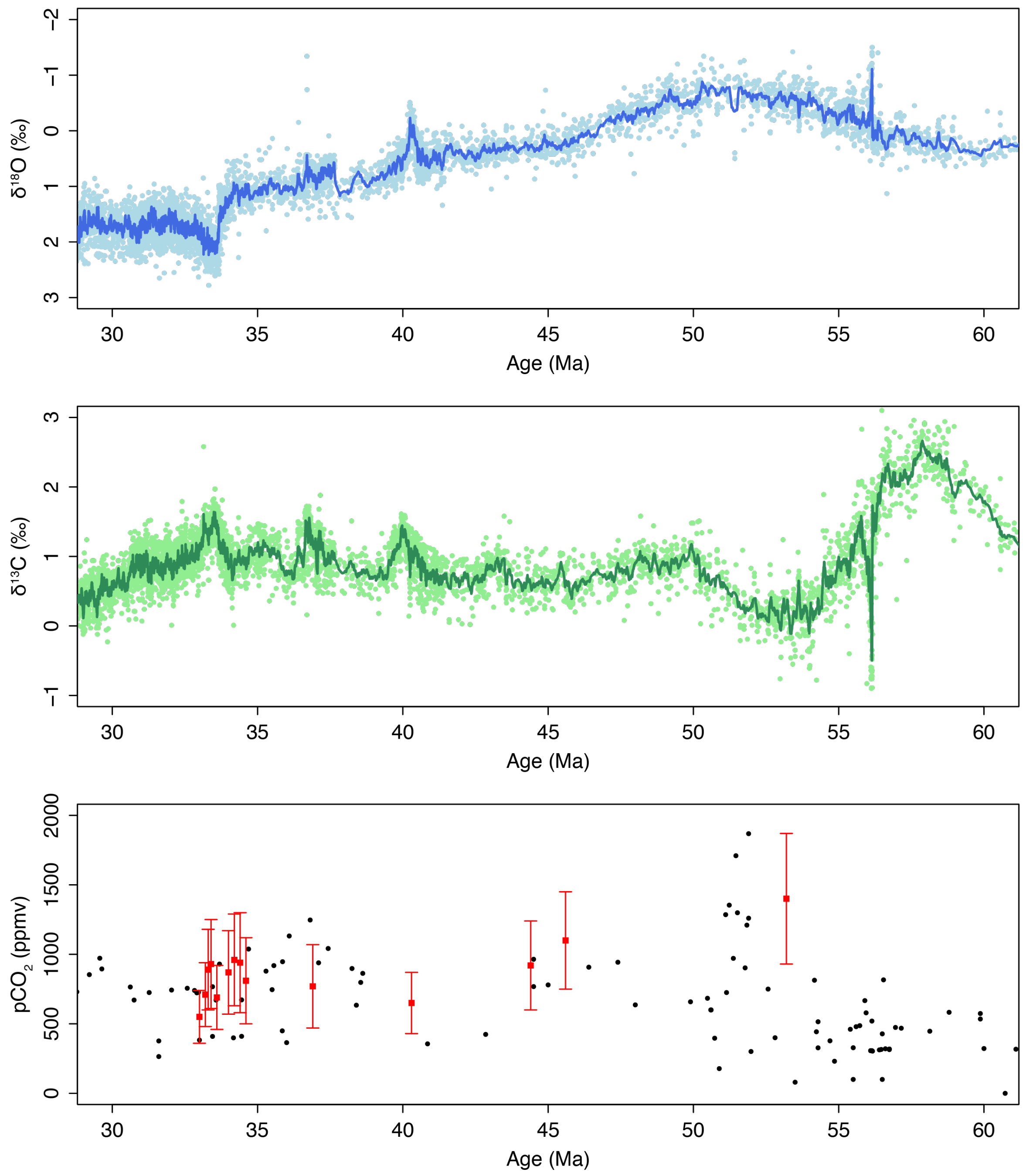

Supplementary Figure 10: Eocene trends in benthic foraminiferal $\delta^{18}O$ and $\delta^{13}C$, and atmospheric pCO_2. a, Benthic $\delta^{18}O$ compilation as published in Cramer et al. (2009)[4], adjusted to the framework of the GTS2012[5] and plotted as individual data points and as a 10-point running average (solid line). **b,** Benthic $\delta^{13}C$ compilation as published in Cramer et al. (2009)[4], adjusted to the framework of the GTS2012[5] and plotted as individual data points and as a 10-point running average (solid line). **c,** Atmospheric pCO_2 compilation as published in Foster et al. (2017)[6], with the $\delta^{11}B$-based pCO_2 estimates of Anagnostou et al. (2016)[7] highlighted in red.

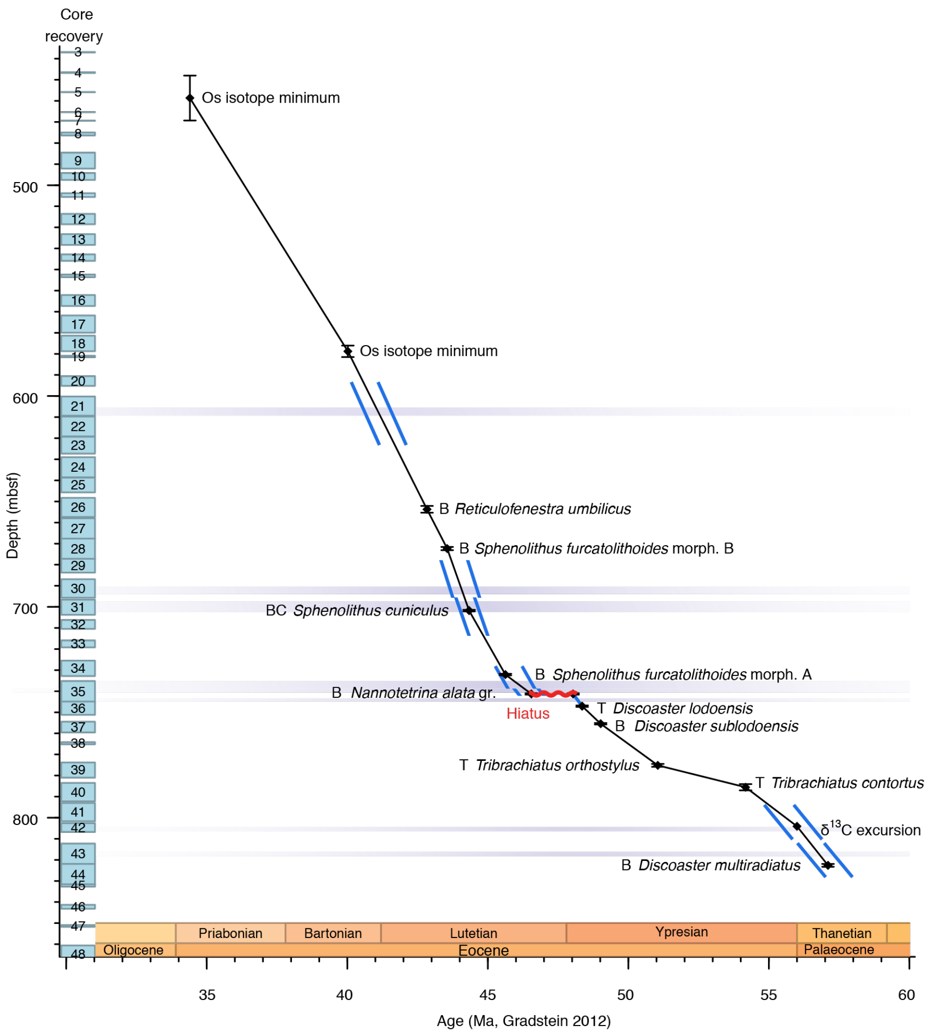

Supplementary Figure 11: Age model for Site 959 Hole D as presented in Cramwinckel et al. (2018)[8]. Diamonds with error bars show calcareous nannofossil and chemostratigraphic tie-points, adjusted to the framework of the GTS 2012[5]. The Os isotope minimum at ~40 Ma is derived from the MECO Os_i records presented in this study.

Supplementary Table 1: Age model for Site 959.

Stratigraphic datum	Depth (mbsf)	Age (Ma) [GTS 2004][9]	Age (Ma) [GTS 2012][5]	Source
$^{187}Os/^{188}Os$ minimum	466.6	34.5	34.65	Ravizza & Paquay (2008)[10]
TEX_{86} highest value at MECO peak	578.25	-	40.06	This study, based on Cramwinckel et al. (2018)[8], Bohaty et al. (2009)[11]
TEX_{86} lowest value at MECO onset	608.84	-	40.52	This study, based on Cramwinckel et al. (2018)[8], Bohaty et al. (2009)[11]
FCO *Reticulofenestra umbilicus*	654.545	-	42.84	Shafik et al. (1998)[12], Cramwinckel et al. (2018)[8]

Supplementary Table 2: Age model for Site 1263.

Stratigraphic datum	Depth (mbsf)	Depth (adj rmcd) [Westerhold et al., 2015][13]	Age (Ma) [Pälike et al., 2006][14]	Age (Ma) [GTS 2012][5]	Source
$\delta^{13}C$ highest value	113.7	135.27	39.2	39.3	Bohaty et al. (2009)[11]
$\delta^{18}O$ final lowest value at MECO peak	119.6	141.29	39.99	40.06	Bohaty et al. (2009)[11]
$\delta^{18}O$ final highest value at MECO onset	122.6	145.83	40.552	40.52	Bohaty et al. (2009)[11]
$\delta^{13}C$ lowest value	124.9	148.13	40.925	40.81	Bohaty et al. (2009)[11]
C18r - C19n boundary	127.85	151.08	41.358	41.154	Pälike et al. (2006)[14]
C19n - C19r boundary	129.25	152.48	41.51	41.39	Pälike et al. (2006)[14]
$\delta^{13}C$ lowest value	131.6	154.8	41.796	41.64	Bohaty et al. (2009)[11]

Supplementary Table 3: Age model for Site U1333.

Stratigraphic datum	Depth (adj rmcd) [Westerhold et al., 2012][15]	Age (Ma) [Pälike et al., 2006][14]	Age (Ma) [GTS 2012][5]	Source
C17r - C18n.1n boundary	157.3	38.449	38.615	Pälike et al. (2006)[14]
T S. obtusus	158.15	38.562	38.71	Toffanin et al. (2013)[16]
T C. grandis	158.55	38.612	38.76	Toffanin et al. (2013)[16]
T C. solitus	159.95	38.788	38.92	Toffanin et al. (2013)[16]
B S. obtusus	164.15	39.314	39.4	Toffanin et al. (2013)[16]
C18n.1n - C18n.1r boundary	166.075	39.554	39.627	Pälike et al. (2006)[14]
C18n.1r - C18n.2n boundary	166.875	39.602	39.698	Pälike et al. (2006)[14]
C18n.2n - C18r boundary	169.57	40.084	40.145	Pälike et al. (2006)[14]
Bc D. hesslandii	172.69	40.421	40.41	Toffanin et al. (2013)[16]
Bc D. bisectus	172.69	40.421	40.41	Toffanin et al. (2013)[16]
T Furcatolithoides	174.5	40.614	40.56	Toffanin et al. (2013)[16]
C18r - C19n boundary	181.5	41.358	41.154	Pälike et al. (2006)[14]
C19n - C19r boundary	183.225	41.51	41.39	Pälike et al. (2006)[14]

Supplementary Table 4: Overview of all LOSCAR model scenarios. All forcings represent a gradual, linear increase/decrease to maximum values from t = 50 kyr to t = 550 kyr and are followed by a sudden drop to initial values. Initial pCO_2 concentrations were set at 750 ppmv in all simulations.

Scenario and Supplementary Figure	Silicate weathering flux	Volcanic CO_2 flux	Carbonate weathering flux
1 (5)	Feedback	+10%	Feedback
2 (5)	Feedback	+15%	Feedback
3 (5)	Feedback	+20%	Feedback
4 (6)	Constant	+10%	Constant
5 (6)	Constant	+15%	Constant
6 (6)	Constant	+20%	Constant
7 (7)	-10%	Constant	Constant
8 (7)	-15%	Constant	Constant
9 (7)	-20%	Constant	Constant
10 (8)	-5%	+5%	Constant
11 (9)	-10%	Constant	-10%
12 (10)	-10%	Constant	Feedback

Supplementary Table 5: Overview of all default Os cycle parameters. Present-day values are taken from the literature or fitted to match the present-day steady state observations. Pre-MECO values are either assumed to be similar to the present-day values or fitted to match the pre-MECO steady state observations.

Parameter	Parameter description	Value	Reference and Comments
Present-day values			
N	Os inventory in oceans	7.2 x 10^7 mol	Peucker-Ehrenbrink & Ravizza (2000)[17]
F_{riv}	Riverine Os flux to oceans	1800 mol/yr	Peucker-Ehrenbrink & Ravizza (2000)[17]
F_{hyd}	Hydrothermal Os flux to oceans	483 mol/yr	Calculated for steady state at R_{sw} = 1.06
F_{ext}	Extraterrestrial Os flux to oceans	80 mol/yr	Peucker-Ehrenbrink & Ravizza (2000)[17]
F_{sed}	Sedimentary Os flux from oceans	2363 mol/yr	Calculated for steady state
R_{riv}	$^{187}Os/^{188}Os$ composition of rivers	1.4	Peucker-Ehrenbrink & Ravizza (2000)[17]
R_{hyd}	$^{187}Os/^{188}Os$ composition of hydrothermal fluids	0.13	Meisel et al. (2001)[18]
R_{ext}	$^{187}Os/^{188}Os$ composition of extraterrestrial dust	0.13	Peucker-Ehrenbrink & Ravizza (2000)[17]
R_{sw}	$^{187}Os/^{188}Os$ composition of seawater	1.06	Peucker-Ehrenbrink & Ravizza (2000)[17]
Pre-MECO values			
N	Os inventory in oceans	7.2 x 10^7 mol	Assumed similar to present-day value
F_{riv}	Riverine Os flux to oceans	865 mol/yr	Calculated for steady state at R_{sw} = 0.55
F_{hyd}	Hydrothermal Os flux to oceans	1418 mol/yr	Calculated for steady state at R_{sw} = 0.55
F_{ext}	Extraterrestrial Os flux to oceans	80 mol/yr	Assumed similar to present-day value
F_{sed}	Sedimentary Os flux from oceans	2363 mol/yr	Assumed similar to present-day value
R_{riv}	$^{187}Os/^{188}Os$ composition of rivers	1.4	Assumed similar to present-day value
R_{hyd}	$^{187}Os/^{188}Os$ composition of hydrothermal fluids	0.13	Assumed similar to present-day value
R_{ext}	$^{187}Os/^{188}Os$ composition of extraterrestrial dust	0.13	Assumed similar to present-day value
R_{sw}	$^{187}Os/^{188}Os$ composition of seawater	0.55	This study

Supplementary References

1. Seton, M. *et al.* Global continental and ocean basin reconstructions since 200Ma. *Earth-Science Rev.* **113,** 212–270 (2012).
2. Torsvik, T. H. *et al.* Phanerozoic polar wander, palaeogeography and dynamics. *Earth-Science Rev.* **114,** 325–368 (2012).
3. Sluijs, A., Zeebe, R. E., Bijl, P. K. & Bohaty, S. M. A middle Eocene carbon cycle conundrum. *Nat. Geosci.* **6,** 429–434 (2013).
4. Cramer, B. S., Toggweiler, J. R., Wright, J. D., Katz, M. E. & Miller, K. G. Ocean overturning since the late cretaceous: Inferences from a new benthic foraminiferal isotope compilation. *Paleoceanography* **24,** 1–14 (2009).
5. Gradstein, F. M., Ogg, J. G., Schmitz, M. & Ogg, G. *The Geologic Time Scale 2012 2-Volume Set*. (Elsevier, 2012).
6. Foster, G. L., Royer, D. L. & Lunt, D. J. Future climate forcing potentially without precedent in the last 420 million years. *Nat. Commun.* **8,** 1–8 (2017).
7. Anagnostou, E. *et al.* Changing atmospheric CO_2 concentration was the primary driver of early Cenozoic climate. *Nature* **533,** 380–384 (2016).
8. Cramwinckel, M. J. *et al.* Synchronous tropical and polar temperature evolution in the Eocene. *Nature* (2018). doi:10.1038/s41586-018-0272-2
9. Gradstein, F. M., Ogg, J. G. & Smith, A. G. *A Geologic Time Scale 2004*. (Cambridge University Press, 2005).
10. Ravizza, G. E. & Paquay, F. S. Os isotope chemostratigraphy applied to organic-rich marine sediments from the Eocene-Oligocene transition on the West African margin (ODP Site 959). *Paleoceanography* **23,** (2008).
11. Bohaty, S. M., Zachos, J. C., Florindo, F. & Delaney, M. L. Coupled greenhouse warming and deep-sea acidification in the middle Eocene. *Paleoceanography* **24,** (2009).
12. Shafik, S., Watkins, D. K. & Shin, I. C. Calcareous Nannofossil Paleogene Biostratigraphy, Côte D'Ivoire-Ghana Marginal Ridge, Eastern Equatorial Atlantic. *Proc. Ocean Drill. Program, Sci. Results* **159,** 413–431 (1998).
13. Westerhold, T., Röhl, U., Frederichs, T., Bohaty, S. M. & Zachos, J. C. Astronomical calibration of the geological timescale: Closing the middle Eocene gap. *Clim. Past* **11,** 1181–1195 (2015).
14. Pälike, H. *et al.* The heartbeat of the Oligocene climate system. *Science* **314,** 1894–

1898 (2006).

15. Westerhold, T. *et al.* Revised composite depth scales and integration of IODP Sites U1331-U1334 and ODP Sites 1218-1220. *Proc. Integr. Ocean Drill. Progr.* **320,** (2012).

16. Toffanin, F., Agnini, C., Rio, D., Acton, G. & Westerhold, T. Middle Eocene to early Oligocene calcareous nannofossil biostratigraphy at IODP Site U1333 (equatorial Pacific). *Micropaleontology* **59,** 69–82 (2013).

17. Peucker-Ehrenbrink, B. & Ravizza, G. E. The marine osmium isotope record. *Terra Nov.* **12,** 205–219 (2000).

18. Meisel, T., Walker, R. J., Irving, A. J. & Lorand, J. P. Osmium isotopic compositions of mantle xenoliths: A global perspective. *Geochim. Cosmochim. Acta* **65,** 1311–1323 (2001).
