# Supplementary material for: Middle Eocene greenhouse warming facilitated by diminished weathering feedback
Source: Nat Commun. 2018 Jul 23;9:2877. doi: 10.1038/s41467-018-05104-9 (PMC6056486; doi:10.1038/s41467-018-05104-9)
Supplement: Supplementary file 2 — Description of Additional Supplementary Files [file 41467_2018_5104_MOESM2_ESM.pdf]

Description of Additional Supplementary Files

File Name: Supplementary Data 1
Description: All Re and Os data.

File Name: Supplementary Data 2
Description: Progressive two-component mixing model involving seawater and basalts.

File Name: Supplementary Software 1
Description: Os cycle model code.

Note: this code makes use of R package deSolve to solve differential equations.
